# Impact of deceased donor with acute kidney injury on subsequent kidney transplant outcomes–an ANZDATA registry analysis

**DOI:** 10.1371/journal.pone.0249000

**Published:** 2021-03-25

**Authors:** Juan Pei, Yeoungjee Cho, Yong Pey See, Elaine M. Pascoe, Andrea K. Viecelli, Ross S. Francis, Carolyn van Eps, Nicole M. Isbel, Scott B. Campbell, Philip A. Clayton, Jeremy Chapman, Michael Collins, Wai Lim, Wen Tang, Germaine Wong, Carmel M. Hawley, David W. Johnson

**Affiliations:** 1 Department of Nephrology, Xiamen University and Fujian Medical University Affiliated First Hospital, Xiamen, China; 2 Department of Nephrology, Princess Alexandra Hospital, Brisbane, Australia; 3 Australasian Kidney Trials Network, Faculty of Medicine, University of Queensland, Brisbane, Australia; 4 Australia and New Zealand Dialysis and Transplant (ANZDATA) Registry, South Australian Health and Medical Research Institute (SAHMRI), Adelaide, Australia; 5 Department of Renal Medicine, Tan Tock Seng Hospital, Novena, Singapore; 6 Department of Medicine, The University of Adelaide, Adelaide, Australia; 7 Central and Northern Adelaide Renal and Transplantation Service, Royal Adelaide Hospital, Adelaide, Australia; 8 Department of Renal Medicine, Westmead Hospital, Western Sydney Local Health District, Sydney, NSW, Australia; 9 Department of Renal Medicine, Auckland City Hospital, Auckland District Health Board, New Zealand; 10 Department of Renal Medicine, Sir Charles Gairdner Hospital, Nedlands, Australia; 11 Department of Nephrology, Peking University Third Hospital, Beijing, China; 12 Translational Research Institute, Brisbane, Australia; Imperial College Healthcare NHS Trust, UNITED KINGDOM

## Abstract

**Background:**

The need for kidney transplantation drives efforts to expand organ donation. The decision to accept organs from donors with acute kidney injury (AKI) can result in a clinical dilemma in the context of conflicting reports from published literature.

**Material and methods:**

This observational study included all deceased donor kidney transplants performed in Australia and New Zealand between 1997 and 2017. The association of donor-AKI, defined according to KDIGO criteria, with all-cause graft failure was evaluated by multivariable Cox regression. Secondary outcomes included death-censored graft failure, death, delayed graft function (DGF) and acute rejection.

**Results:**

The study included 10,101 recipients of kidneys from 5,774 deceased donors, of whom 1182 (12%) recipients received kidneys from 662 (11%) donors with AKI. There were 3,259 (32%) all-cause graft failures, which included 1,509 deaths with functioning graft. After adjustment for donor, recipient and transplant characteristics, donor AKI was not associated with all-cause graft failure (adjusted hazard ratio [HR] 1.11, 95% CI 0.99–1.26), death-censored graft failure (HR 1.09, 95% CI 0.92–1.28), death (HR 1.15, 95% CI 0.98–1.35) or graft failure when death was evaluated as a competing event (sub-distribution hazard ratio [sHR] 1.07, 95% CI 0.91–1.26). Donor AKI was not associated with acute rejection but was associated with DGF (adjusted odds ratio [OR] 2.27, 95% CI 1.92–2.68).

**Conclusion:**

Donor AKI stage was not associated with any kidney transplant outcome, except DGF. Use of kidneys with AKI for transplantation appears to be justified.

## Introduction

Kidney transplantation is an important treatment for end-stage kidney disease (ESKD) since individuals receiving kidney transplants generally have better survival and quality of life compared with those who remain on dialysis [1–3]. Given that the availability of suitable donor kidneys is limited and insufficient to meet the needs of the increasing numbers of patients with ESKD, there have been attempts in recent years to expand the criteria for donor kidneys that are considered potentially suitable for transplantation [4].

One such consideration is deceased donors with acute kidney injury (AKI). In a recent study from the United States, AKI defined by Acute Kidney Injury Network criteria occurred in up to 24% of all deceased donors and could potentially contribute significantly to the pool of kidneys offered for transplantation [5]. However, while AKI is generally expected to recover with time, many clinicians and patients hesitate to accept kidneys from donors with AKI for fear of poor outcomes, leading to difficulties and delays with allocation and sometimes to these kidneys ultimately being discarded [6–8]. Several retrospective cohort studies have suggested that AKI is associated with an increased risk of DGF [8, 9], although the findings in relation to long-term kidney transplant outcomes, such as graft survival, have been conflicting [5, 7, 10–12].

Therefore, this study aimed to investigate the associations between donor AKI and recipient transplant outcomes using the Australia and New Zealand Dialysis and Transplant (ANZDATA) Registry and Organ Donation (ANZOD) Registry.

## Materials and methods

Research using ANZDATA and ANZOD Registry data was approved by the Princess Alexandra Hospital Human Research Ethics Committee (LNR/2019/QMS/51797) and the ANZDATA executive (02/04/2019). This manuscript was prepared in accordance with the Strengthening The Reporting of Observational studies in Epidemiology (STROBE) guidelines [13].

### Study population

This retrospective, observational cohort study included all deceased donors (adult or pediatric) and their kidney transplant recipients (adult or pediatric) in Australia and New Zealand from 1 January 1997 to 31 December 2017. Recipients of multi-organ or repeat kidney transplants during this study period were excluded. Donors with missing admission or terminal serum creatinine (SCr) data were not included as their AKI status could not be confirmed.

### Exposure factor

Donor AKI was defined according to the Kidney Disease Improving Global Outcomes (KDIGO) criteria (excluding urine output criteria due to incomplete data on patients) as follows: an increase in SCr level by ≥0.3mg/dL (26.5μmol/L) within 48 h or an increase in a SCr level to ≥1.5 times from time of donor admission to time of organ procurement [14]. The stages of AKI were defined according to the severity of increase in SCr level: stage 1, ≥0.3 mg/dL (26.5μmol/L) or 1.5–1.9 times baseline increase; stage 2, 2–2.9 times; and stage 3, ≥3.0 times baseline or increase in SCr to ≥354μmol/L or decrease in estimated glomerular filtration rate (eGFR) to <35 ml/min per 1.73 m^2^ in patients under the age of 18 years [14].

### Data collection

The deceased donor demographic variables extracted were age, race, gender, smoking status, height, weight, hypertension, diabetes mellitus, cause of death, expanded-criteria donor (ECD) [15], standard-criteria donor (SCD), donation after circulatory death (DCD) donors, kidney donor risk index (KDRI) [16], kidney donor profile index (KDPI) [17], procurement biopsy performed (yes/no), number of transplanted kidneys from the source donor (1 or 2), and hepatitis C seropositivity. Treatment details included admission and terminal SCr concentration, admission and terminal serum urea concentration, terminal eGFR (estimated by the modification of diet in renal disease [MDRD] formula) [18], terminal urine output, and presence and duration of oliguria (defined as urine output <20 mL/hr) in the last 12 hours. The recipient and transplantation demographic variables extracted were age, race, gender, smoking status, height, weight, cause of ESKD, comorbid conditions at time of transplantation (diabetes mellitus, chronic lung disease, coronary artery disease, peripheral vascular disease, and cerebrovascular disease), prior kidney replacement therapy modality (nil [i.e. pre-emptive], peritoneal dialysis, hemodialysis, kidney transplant), dialysis duration (months), previous kidney transplant prior to study period, number of human leukocyte antigen mismatches, panel reactive antibody, body mass index at time of transplant, total ischemia time, induction immunosuppression regimen, transplant era and transplant country.

### Outcomes

The primary outcome of this study was all-cause graft failure (defined as return to dialysis, re-transplantation or death with functioning graft). Secondary outcomes included death-censored graft failure, all-cause mortality (excluding death occurring beyond 30 days of graft failure), acute rejection within 6 months after transplantation (defined as either biopsy-proven rejection or clinical rejection episodes [without biopsy] requiring treatment), and delayed graft function (DGF—defined as requiring dialysis within 72 hours [prior to 2017] or 7 days of post-transplantation [from 2017]). Data were censored as of 31st December 2017.

### Statistical analysis

Recipient, donor and transplant characteristics are expressed as number (percentage) for categorical variables, mean ± standard deviation for normally distributed continuous variables, and median (interquartile range) for continuous variables that were not normally distributed. Comparisons of characteristics between kidney transplants recipients who received a donor kidney with AKI and those who received a donor kidney without AKI were performed by Chi-square or Fisher’s exact test, as appropriate, for categorical variables. Student’s t-test was performed for normally distributed continuous variables, and Mann-Whitney test or Kruskal-Wallis test for continuous variables that were not normally distributed. Time to event outcomes (all-cause graft failure, death-censored graft failure, all-cause mortality) by donor AKI status were displayed using Kaplan-Meier survival curves and compared with log-rank tests. Three multivariable Cox proportional hazards regression models using robust standard errors were subsequently constructed using variables with p<0.2 on univariable analyses in addition to variables deemed to be clinically important (donor weight and donor ethnicity) [16]. Variables with ≥5% missing data were excluded from the multivariable model. Therefore Model 1 included donor AKI (yes/no) and the component factors of KDRI (age, height, weight, ethnicity, history of hypertension, diabetes, cause of death, hepatitis C seropositivity, and DCD, but excluded terminal SCr as this was already included as an AKI covariate). Model 2 included the Model 1 variables with additional donor factors (sex, number of kidneys eventually donated). Model 3 included the Model 2 variables with additional recipient and transplant factors (age, sex, ethnicity, body mass index [BMI], previous transplant, pre-emptive transplant, cause of ESKD, dialysis vintage, induction immunosuppression, number of human leukocyte antigen-mismatches [HLA-A, HLA-B, HLA-DR], peak panel reactive antibody, total ischemia time, era [1997–2003, 2004–2010, 2011–2017]). Proportional hazards assumption of Cox model was checked graphically and by Schoenfeld residuals. Competing risk analysis was also conducted for graft failure with death as a competing event using the method of Fine and Gray [19]. The sub-distributional hazard ratio (sHR) and 95% confidence interval (CI) were calculated to estimate the covariate effects on the cumulative incidence. Covariates included in the competing risk models were identical to those included in the Cox regression models. Logistic regression was performed to evaluate association between AKI status and development of DGF and acute rejection. There were no missing data for acute rejection within 6 months. There were missing data for DGF in a small proportion (1.8%) of cases; these were assumed to be absent (i.e. no DGF) in the logistic regression analysis. Clustering due to paired kidneys from the same donor was accounted for by using robust standard errors [20]. The relationships between AKI stage (1, 2 or 3) and the primary and secondary transplant outcomes were also evaluated. The first-order interactions between donor-AKI and donor and recipient factors were examined in each multivariable model to assess their effect modification. Statistically significant interaction terms were included in the models. All data were analyzed using Stata/SE14.0 (College Station, TX). P values < 0.05 were considered statistically significant.

## Results

### Study population

**Fig 1:**
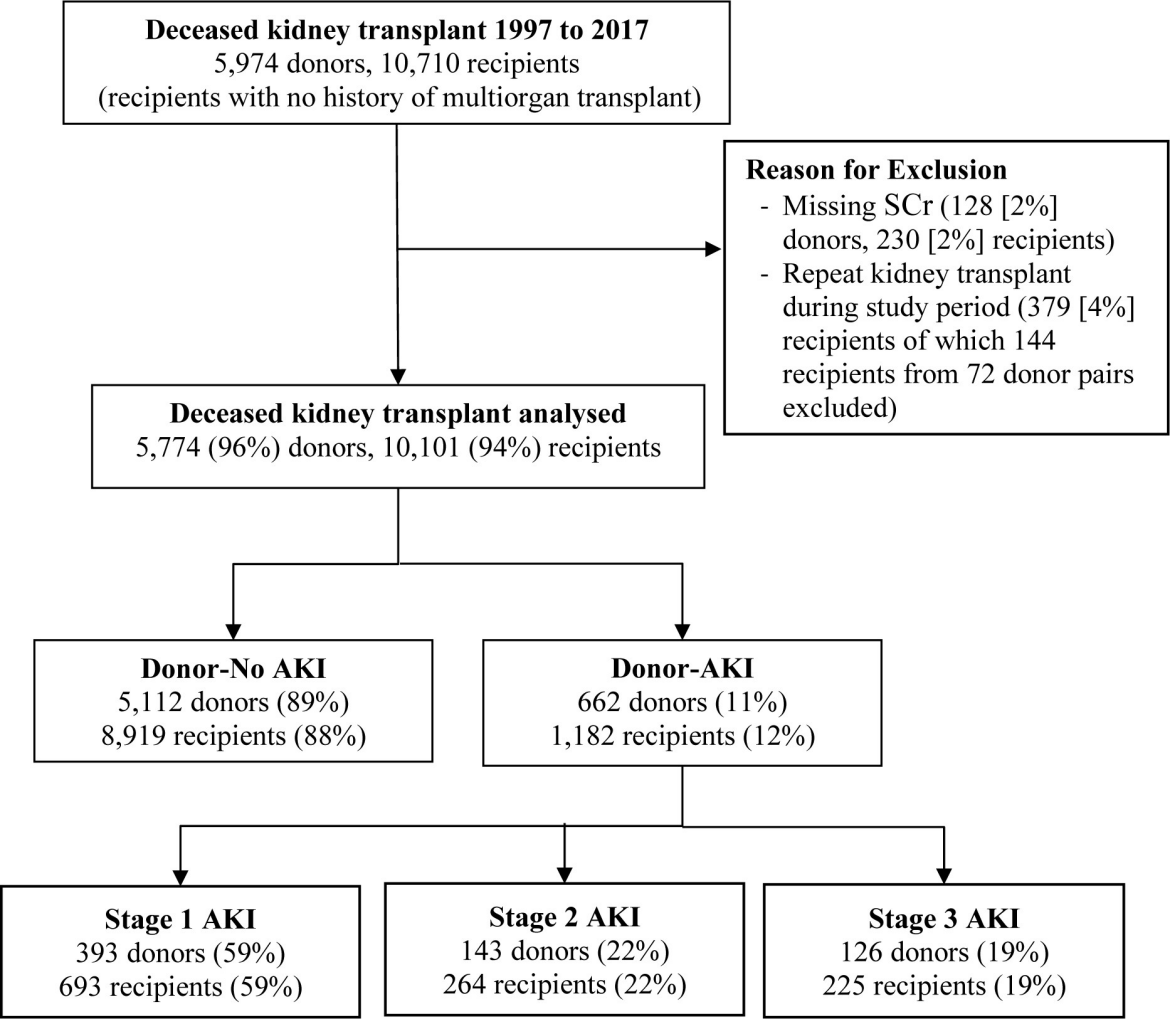
Flow chart for kidney transplant recipients included in study.

During the study period, 10,710 patients with ESKD received kidney transplants from 5,974 deceased donors. 609 kidney transplant recipients and 200 donors were excluded from further analysis due to missing data or because they received a repeat kidney transplant during the analysis period (Fig 1). Therefore, a total of 5,774 donors and 10,101 recipients were included in the analysis. Overall missingness of data was less than 5% for all variables (except for procurement biopsy performed 10%).

### Comparisons of donor and recipient characteristics according to AKI status

**Fig 2:**
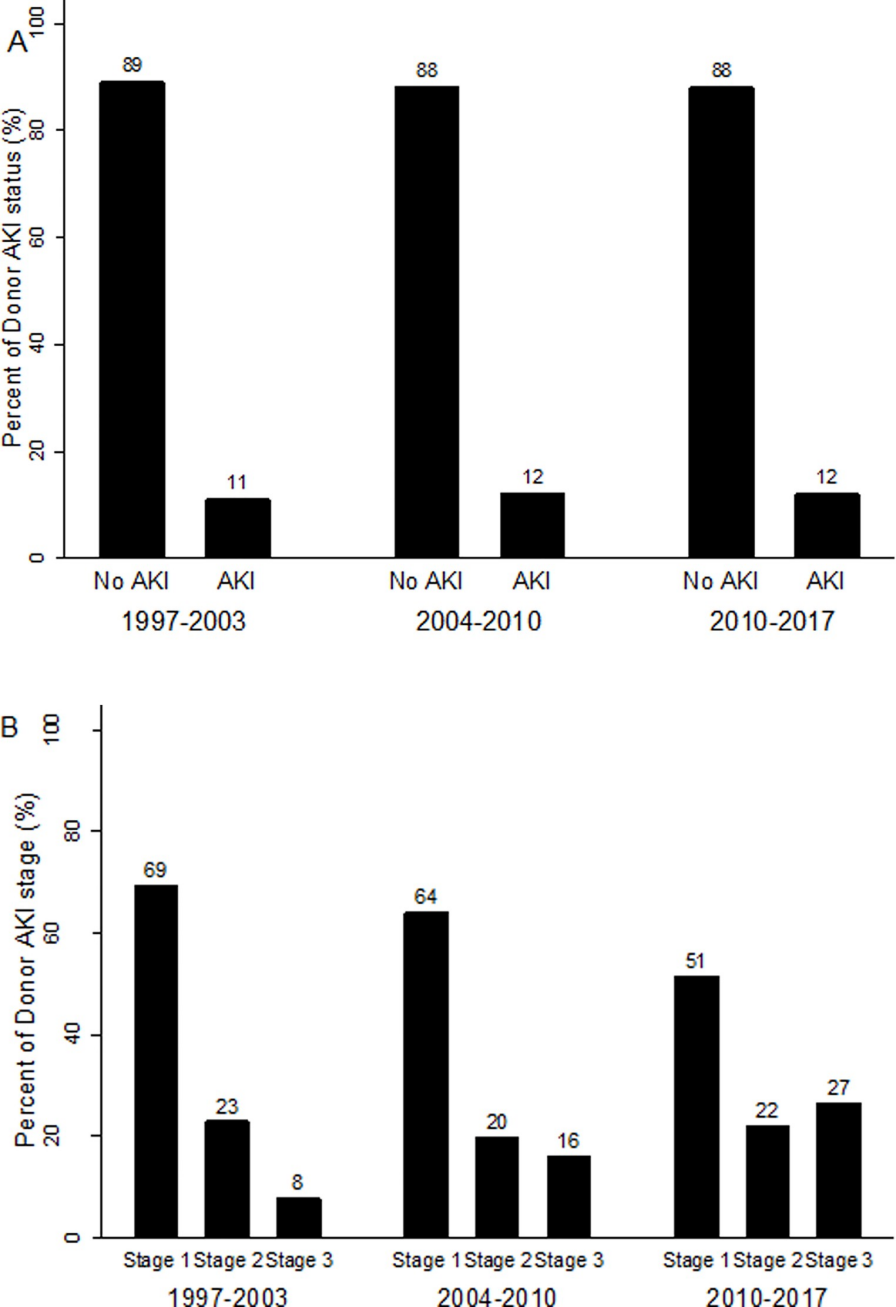
(A) Histogram showing the percentage of donor AKI status by Era. (B) Histogram showing the percentage of donor AKI stage by Era.

Of 10,101 recipients, 1,182 (12%) transplant recipients received kidneys from 662 donors with AKI; 693 recipients (7%) with stage 1, 264 (3%) with stage 2, and 225 (2%) with stage 3. Of 662 donors with AKI, 91 donors donated both kidneys to 91 individual recipients, 51 only donated one kidney while the remaining 520 donors donated both kidneys to 1040 separate recipients. Table 1 shows the comparisons of donor characteristics by donor AKI status. Compared with donors without AKI, donors with AKI were more likely to be male, have a higher weight, a record of anoxia as the cause of death, a procurement kidney biopsy, a shorter admission duration prior to kidney procurement and a higher likelihood of donating only one kidney instead of two. There were no differences between the two groups regarding age, ethnicity, height, diabetes, hypertension, DCD status and era (Fig 2A). Characteristics of donors listed by different severity stages of AKI are shown in S1 Table. Donors with stage 3 AKI were younger, had lower KDPI and KDRI values, were less likely to be hypertensive or be ECD, and were more likely to have a procurement biopsy, have anoxia as the main cause of AKI, and have donated in a more recent era (1997–2003: 8%, 2004–2010: 16%, 2011–2017: 27%, Fig 2B). Other donor characteristics, including sex, ethnicity, weight, height, diabetes and DCD status, were comparable across the AKI stages.

**Table 1:** Baseline donor characteristics according to donor AKI status.

Variable	All	No AKI	AKI	P value
	(N = 5774)	(N = 5112)	(N = 662)	
Age, years	46 (30, 58)	46 (30, 58)	46 (30, 58)	0.74
Male	3257(56%)	2847(56%)	410(62%)	<0.01
Ethnicity origin^#^				0.50
Caucasoid	5266 (91%)	4655 (91%)	611 (92%)	
Aboriginal/Torres Str	69 (1%)	62 (1%)	7 (1%)	
Asian	257 (5%)	226 (5%)	31 (5%)	
Māori	77 (1%)	71 (1%)	6 (1%)	
Islander	28 (1%)	25 (1%)	3 (<1%)	
Other	77 (1%)	73 (1%)	4 (1%)	
Weight, kg	75 (65, 87)	75 (65, 86)	80 (68, 90)	<0.01
Height, cm	171 (164, 180)	170 (164, 180)	172 (165, 180)	0.11
Diabetes	313 (5%)	270 (5%)	43 (7%)	0.20
Hypertension*	1239 (22%)	1090 (22%)	149 (23%)	0.46
Smoking				0.49
Current	2211 (38%)	1957 (38%)	254 (38%)	
Former	1107 (19%)	983 (19%)	124 (19%)	
Never	2392 (41%)	2119 (42%)	273 (41%)	
Unknown	64 (1%)	53 (1%)	11 (2%)	
Cause of death^#^				<0.01
Head trauma	1333 (23%)	1203 (24%)	130 (20%)	
Anoxia	1223 (21%)	991 (19%)	232 (35%)	
Cerebrovascular/stroke	2817 (49%)	2557 (50%)	260 (39%)	
CNS tumor	58 (1%)	55 (1%)	3 (1%)	
Other	343 (6%)	306 (6%)	37 (6%)	
Donor circulatory death	826 (14%)	727 (14%)	99 (15%)	0.61
Hepatitis C seropositive^#^	37 (1%)	36 (1%)	1 (<1%)	0.12
ECD	1565 (27%)	1357 (27%)	208 (32%)	<0.01
KDRI*	1.2 (1.0, 1.6)	1.2 (0.9, 1.5)	1.3 (1.1, 1.7)	<0.01
KDPI, %*	53 (27, 77)	51 (25, 76)	62 (37.5, 84)	<0.01
Admission to procurement, days	2.4 (1.6, 4.1)	2.4 (1.6, 4.1)	2.1 (1.5, 3.8)	<0.01
Admission SCr, μmol/L	80 (65, 102)	80 (65, 101)	85 (64, 111)	0.01
Terminal SCr, μmol/L	73 (59, 97)	70 (56, 89)	163 (110, 250)	<0.01
Terminal urine output, ml/kg/h	1.1 (0.6, 1.8)	1.1 (0.7, 1.9)	0.9 (0.5, 1.5)	<0.01
Oliguria last 12 hours (<20mls/h)	669 (12%)	521 (10%)	148 (22%)	<0.01
Oliguria duration, hours	2 (1, 4)	2 (1, 3)	3 (1, 7)	<0.01
Procurement biopsy performed*	770 (15%)	606 (13%)	164 (28%)	<0.01
Number of individual kidneys transplanted				<0.01
1	291 (5%)	240 (5%)	51 (8%)	
2	5483 (95%)	4872 (95%)	611 (92%)	
Era				0.98
1997–2003	1386 (24%)	1229 (24%)	157 (23%)	
2004–2010	1658 (29%)	1466 (29%)	192 (29%)	
2011–2017	2730 (47%)	2417 (47%)	313 (47%)	

Results are presented as medians (interquartile range) or frequency (percentage). Mann-Whitney test for continuous variables. Chi-square or Fisher’s exact test for categorical variables.

Abbreviations: AKI, acute kidney injury; DCD, donation after cardiovascular determination of death; ECD, expanded-criteria donor; KDPI, kidney donor profile index; KDRI, kidney donor risk index; SCr, serum creatinine.

*All variables missing were <1%, except hypertension 1%, KDRI, KDPI 2%, Procurement biopsy performed 10%.

^#^Using Fisher’s exact test.

Recipients of kidneys from donors with AKI were more likely to have diabetes mellitus, be transplanted in Australia (vs. New Zealand), and be on hemodialysis prior to transplantation (Table 2). Post-transplantation, recipients of kidneys with AKI were more likely to have received T cell or B cell depletion therapy. No differences between the two groups were found for dialysis duration before transplant, total ischemia time, HLA mismatch level and era (1997–2003, 2004–2010, 2011–2017).

**Table 2:** Recipient and transplant-related characteristics according to donor AKI status.

Variable	ALL	No AKI	AKI	P value
	(N = 10101)	(N = 8919)	(N = 1182)	
Age, years	51 (41, 60)	51 (41, 60)	51 (41, 60)	0.51
Male	6387 (63%)	5624 (63%)	763 (65%)	0.32
Ethnicity origin				0.52
Caucasoid	7613 (75%)	6730 (75%)	883 (75%)	
Aboriginal/Torres Str	455 (5%)	399 (4%)	56 (5%)	
Asian	1189 (12%)	1042 (12%)	147 (12%)	
Māori	248 (2%)	227 (3%)	21 (2%)	
Islander	290 (3%)	256 (3%)	34 (3%)	
Other	306 (3%)	265 (3%)	41 (3%)	
Height, cm*	170 (162, 177)	170 (162, 177)	170 (160, 177)	0.61
Weight, kg*	75 (64, 87)	75 (64, 87)	75 (64, 87)	0.69
BMI, kg/m^2^*	26 (23, 30)	26 (23, 30)	26 (23, 30)	0.44
Cause of ESKD				0.62
Diabetes	1223 (12%)	1067 (12%)	156 (13%)	
Glomerulonephritis	4454 (44%)	3943 (44%)	511 (43%)	
Hypertension	607 (6%)	539 (6%)	68 (6%)	
Polycystic Disease	1343 (13%)	1173 (13%)	170 (14%)	
Reflux Nephropathy	811 (8%)	718 (8%)	93 (8%)	
Other	1663 (17%)	1479 (17%)	184 (16%)	
Comorbidities				
Chronic lung disease	791 (8%)	696 (8%)	95 (8%)	0.78
Coronary artery disease	1840 (18%)	1613 (18%)	227 (19%)	0.36
Peripheral vascular disease	973 (10%)	854 (10%)	119 (10%)	0.60
Cerebrovascular disease	575 (6%)	509 (6%)	66 (6%)	0.86
Diabetes	1797(18%)	1560 (18%)	237 (20%)	0.03
Graft number				0.18
1	9029 (89%)	7954(89%)	1075(91%)	
2	909 (9%)	817 (9%)	92 (8%)	
≥3	163 (2%)	148 (2%)	15 (2%)	
Transplant Country				<0.01
Australia	8926 (88%)	7831 (88%)	1095 (93%)	
New Zealand	1175 (12%)	1088 (12%)	87 (7%)	
Previous transplant	1072 (10%)	965 (10%)	107 (10%)	0.06
Dialysis duration, months	40 (21, 73)	40 (20, 73)	40 (22, 72)	0.63
Pre-emptive transplant	80 (1%)	73 (1%)	7 (1%)	0.41
Last treatment before transplant				0.04
HD	7235 (71%)	6352 (71%)	883 (75%)	
PD	2786 (28%)	2494 (28%)	292 (24%)	
Pre-emptive transplant	80 (1%)	73 (1%)	7 (1%)	
Total ischemia time, hours*	12 (9, 16)	12 (9, 16)	13 (10, 16)	0.06
HLA mismatch level	3 (2, 5)	3 (2, 5)	4 (2, 5)	0.63
Induction immunosuppression				
Cd25	6602 (65%)	5811 (65%)	791 (67%)	0.23
T cell depletion	659 (7%)	532 (6%)	127 (11%)	<0.01
B cell depletion	22 (<1%)	14 (<1%)	8 (1%)	<0.01
Ivig	124 (1%)	105 (1%)	19 (2%)	0.21
Eculizumab^#^	12 (<1%)	10 (<1%)	2 (<1%)	0.64
Peak PRA (%)*	3 (0, 16)	3 (0, 16)	3 (0, 15)	0.71
Era				0.43
1997–2003	2506 (25%)	2231 (25%)	275 (23%)	
2004–2010	2881 (29%)	2537 (28%)	344 (29%)	
2011–2017	4714 (47%)	4151 (47%)	563 (48%)	

Results are presented as medians (interquartile range) or frequency (percentage). Mann-Whitney test for continuous variables. Chi-square or Fisher’s exact test for categorical variables.

Abbreviations: AKI, acute kidney injury; ESKD, End stage kidney disease; HD, hemodialysis; PD, peritoneal dialysis; HLA, human leukocyte antigen; Ivig, intravenous immunoglobulin; PRA, panel reactive antibody

*All variables missing were <1%, except Height 2%, Weight 2%, BMI 4%, Total ischemia time 4%, Peak PRA 1%.

^#^Using Fisher’s exact test.

### All-cause graft failure

**Fig 3:**
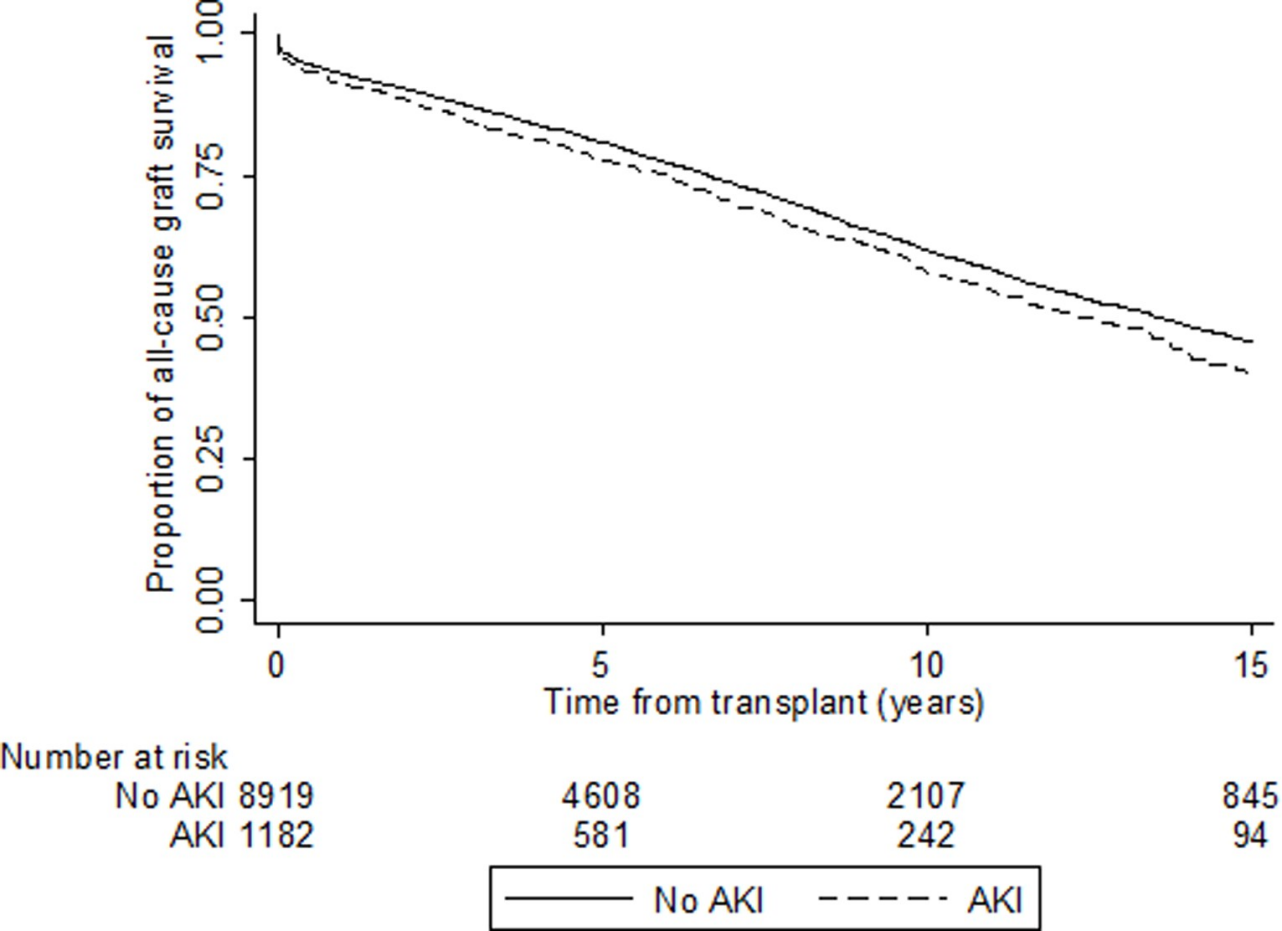
Unadjusted Kaplan-Meier survival curve for all-cause graft survival according to donor acute kidney injury status in kidney transplant recipients in Australia and New Zealand 1997–2017 (HR 1.14, 95% CI 1.01–1.28).

The total follow-up period of the cohort was 64,682 patient-years with a median follow-up period of 5.2 years (interquartile range 2.0–9.6 years). There were 3,259 (32%) all-cause graft failures, which included 1,509 deaths with a functioning graft. Respective 1-year graft survival rates were 93% (95% CI 92–93%) for donors without AKI vs 91% (95% CI 89–93%) from donors with AKI, 5-year graft survival rates were 81% (95% CI 80–82%) for donors without AKI vs 78% (95% CI 75–80%) from donors with AKI, whilst 10-year graft survival rates were 62% (95% CI 60–63%) vs 58% (95% CI 54–62%). Compared with recipients of kidneys without donor AKI, recipients of kidneys with donor AKI experienced a higher unadjusted hazard of all-cause graft failure (HR 1.14, 95% Cl 1.01–1.28; Fig 3, S2 Table). Using multivariable Cox proportional hazards model analysis, donor AKI was associated with a small increased hazard of graft failure in Model 1 (HR 1.15, 95% CI 1.02–1.29) and Model 2 (HR 1.14, 95% CI 1.01–1.28). However, in Model 3, whilst the effect estimate was similar for donor AKI, the association was no longer statistically significant (HR 1.11, 95% CI 0.99–1.26) (Table 3). Recent era was associated with a lower risk of all-cause graft failure (1997–2003 Reference; 2004–2010 HR 0.83, 95% CI 0.75–0.91; 2011–2017 HR 0.61, 95% CI 0.53–0.70). There was no evidence of significant two-way interaction.

**Table 3:** Associations between donor AKI status with all-cause graft failure, death-censored graft failure, all-cause mortality and graft failure with death as a competing event.

Events	Failures	Event rate per 1000 patient-years (95% Cl)	Unadjusted		Model 1		Model 2		Model 3	
			HR (95% Cl)	P value	HR (95% Cl)	P value	HR (95% Cl)	P value	HR (95% Cl)	P value
All-cause graft failure*										
No AKI (n = 8919)	2857	49.7 (47.9–51.5)	Ref		Ref		Ref		Ref	
AKI (n = 1182)	402	56.4 (51.1–62.1)	1.14 (1.01–1.28)	0.03	1.15 (1.02–1.29)	0.02	1.14 (1.01–1.28)	0.03	1.11 (0.99–1.26)	0.08
Death-censored graft failure*										
No AKI (n = 8919)	1540	26.8 (25.4–28.1)	Ref		Ref		Ref		Ref	
AKI (n = 1182)	210	29.4 (25.7–33.7)	1.10 (0.94–1.28)	0.24	1.12 (0.95–1.31)	0.17	1.11 (0.94–1.30)	0.21	1.09 (0.92–1.28)	0.32
All-cause mortality*										
No AKI (n = 8919)	1405	24.4 (23.2–25.7)	Ref		Ref		Ref		Ref	
AKI (n = 1182)	203	28.5 (24.8–32.7)	1.17 (1.01–1.37)	0.04	1.19 (1.01–1.39)	0.03	1.18 (1.01–1.38)	0.04	1.15 (0.98–1.35)	0.09
sHR (95% CI) sHR (95% CI) sHR (95% CI) sHR (95% CI)										
Graft failure with death as a competing event^#^										
No AKI (n = 8919)	1540	26.8 (25.5–28.1)	Ref		Ref		Ref		Ref	
AKI (n = 1182)	210	29.4 (25.7–33.7)	1.07 (0.92–1.24)	0.39	1.09 (0.93–1.28)	0.27	1.08 (0.92–1.26)	0.33	1.07 (0.91–1.26)	0.42

Data presented as HR with 95% confidence interval (95%Cl) from Cox regression models (* for all-cause graft failure, death-censored graft failure and all-cause mortality) or as sub-distribution (sHR) with 95%CI from competing risk model ^(#^ for graft loss with death as a competing event).

Model 1: AKI (KDIGO definition) + KDRI components except for terminal SCr (already considered for the AKI covariate), Model 2: Model 1 + other donor factors such as sex, number of kidneys eventually donated, Model 3: Model 2 + recipient and transplant factors, which included age, sex, ethnicity, body mass index [BMI], previous transplant, pre-emptive transplant, cause of ESKD, dialysis vintage, induction immunosuppression, number of human leukocyte antigen-mismatches [HLA-A, HLA-B, HLA-DR], peak panel reactive antibody, total ischemia time, era [1997–2003, 2004–2010, 2011–2017]

AKI, acute kidney injury; HR, hazard ratio; Cl, confidence interval; sHR, sub-distribution hazard ratio.

### Death-censored graft failure

**Fig 4:**
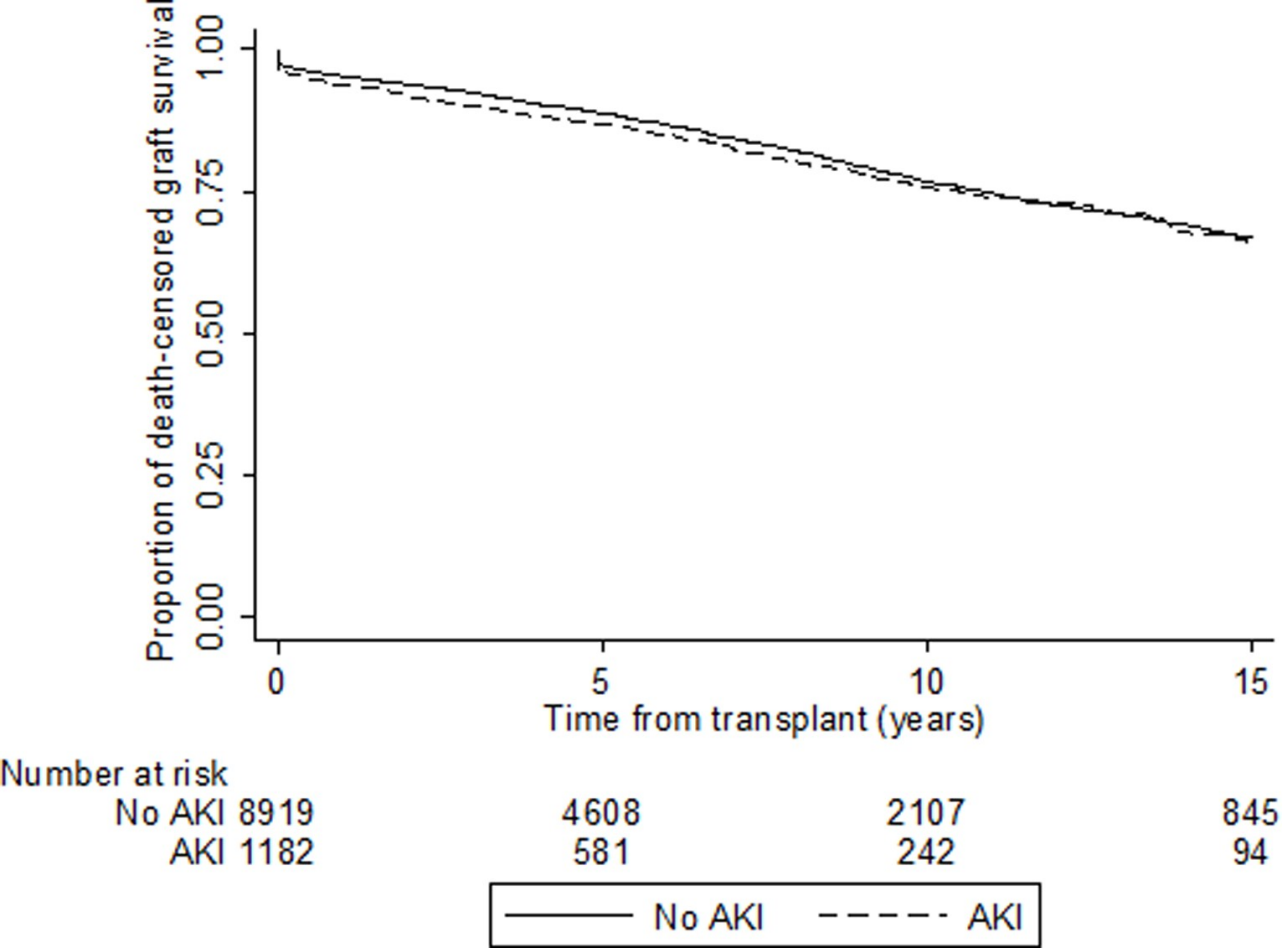
Unadjusted Kaplan-Meier survival curve for death-censored graft survival according to acute kidney injury status in first kidney transplant recipients in Australia and New Zealand 1997–2017 (HR 1.10, 95% CI 0.94–1.28).

Donor AKI was not associated with death-censored graft failure in univariable (HR 1.10, 95% CI 0.94–1.28, Table 3, Fig 4, S2 Table) and multivariable models (Model 1: HR 1.12, 95% CI 0.95–1.31, Model 2: HR 1.11, 95% CI 0.94–1.30 and Model 3: HR 1.09, 95% CI 0.92–1.28, Table 3). Similar results were found when death with a functioning graft was considered as a competing event (unadjusted model: sub-distribution HR [sHR] 1.07, 95% CI 0.92–1.24, Model 1: sHR 1.09, 95% CI 0.93–1.28, Model 2: sHR 1.08, 95% CI 0.92–1.26 and Model 3: sHR 1.07, 95% CI 0.91–1.26, Table 3, S1 Fig). There was no evidence of significant two-way interaction terms.

### All-cause mortality

**Fig 5:**
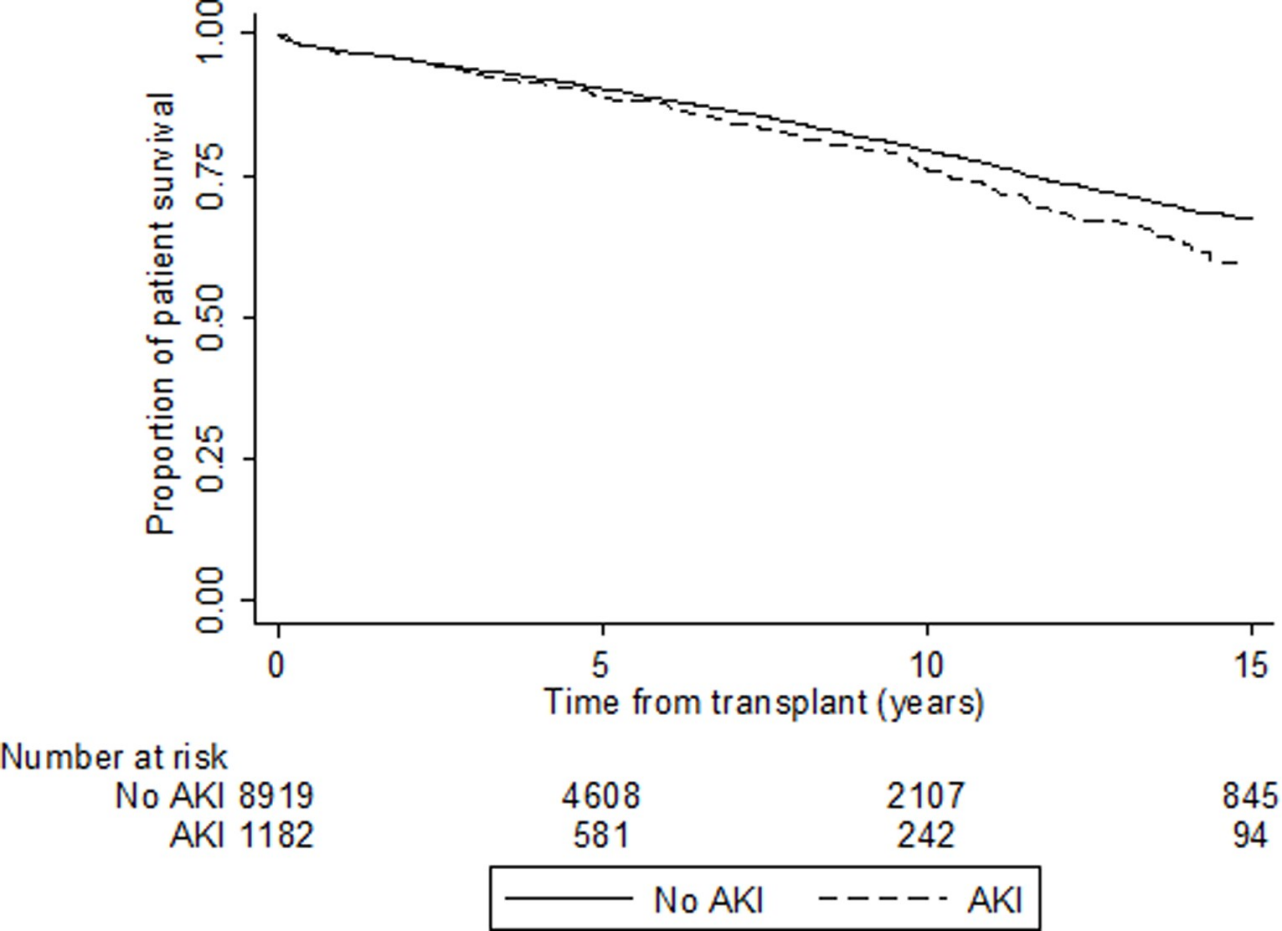
Unadjusted Kaplan-Meier survival curve for patient survival according to donor acute kidney injury status (HR 1.17, 95% CI 1.01–1.37).

A total of 1,608 (16%) transplant recipients died during the study period including 1509 deaths with functioning graft and 99 deaths within 30 days of graft failure. The most common cause of death was cardiovascular disease (31%), followed by malignancy (27%), sepsis/ infection (20%) and other/unknown (23%). There were no differences in causes of death between the two groups. Respective 5-year patient survival rates were 91% (95% CI 90- f91%) for donors without AKI vs. 89% for donors with AKI (95% CI 87–91%), whilst 10-year patient survival rates were 79% (95% CI 78–81%) vs 76% (95% CI 72–79%). Compared with recipients of kidneys without donor AKI, recipients of kidneys with donor AKI experienced a higher unadjusted hazard of mortality (HR 1.17, 95% Cl 1.01–1.37, Fig 5, S2 Table). Using multivariable Cox proportional hazards model analysis, donor AKI was still associated with an increased hazard of mortality in Model 1 (HR 1.19, 95% CI 1.01–1.39) and Model 2 (HR 1.18, 95% CI 1.01–1.38), while in Model 3 the estimate of effect was similar but the association was no longer statistically significant (HR 1.15, 95% CI 0.98–1.35; Table 3). There was no evidence of significant two-way interaction terms.

**Fig 6:**
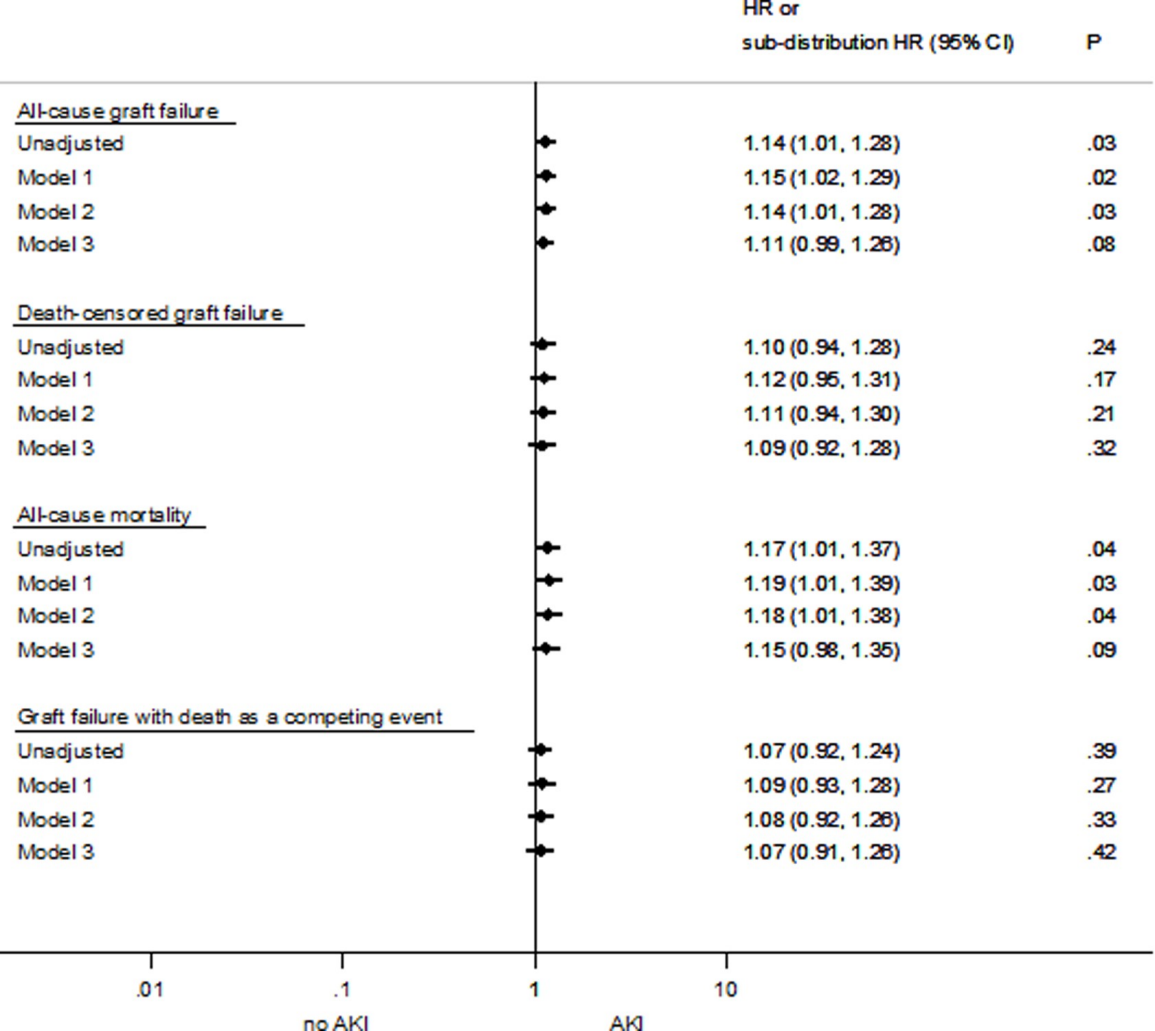
Forest plots for all-cause graft failure, death-censored graft failure, all-cause mortality and graft loss with death as competing event according to acute kidney injury status in kidney transplant recipients in Australia and New Zealand 1997–2017.

In addition to the summary data in Table 3, the association of donor AKI as a binary variable with all outcomes in all presented models is also displayed in a forest plot in Fig 6.

### Early transplant outcomes

#### Delayed graft function

A total of 2,798 (28%) recipients experienced DGF during the follow-up time. Of these, 523 (44%) were from 1,182 donors with AKI and 2275 (26%) were from 8,919 donors without AKI. The fully adjusted OR for DGF among recipients with AKI donor kidneys was 2.27 (95% CI 1.92–2.68, S3 Table).

#### Acute rejection

A total of 2,016 (20%) recipients experienced acute rejection within 6 months after transplantation. Of these, 243 (21%) were from 1,182 donors with AKI and 1,773 (20%) were from 8,919 donors without AKI. The fully adjusted OR for acute rejection among recipients with AKI donor kidneys was 1.06 (95% CI 0.88–1.28, S3 Table).

### Association of AKI stage with transplant outcomes

Stage 1 donor AKI was consistently associated with an increased risk of all-cause graft failure in the unadjusted and multivariable models, although the overall p value for Model 3 was not statistically significant (S4 Table, S2 Fig). No such association was observed for stage 2 and 3 donor AKI (S4 Table).

Similar results were observed between AKI stage and all-cause mortality in the unadjusted and multivariable models (S4 Table, S3 Fig).

No significant association was observed between AKI stage and death-censored failure or graft failure with death as a competing event in the unadjusted and multivariable models (S4 Table, S4 and S5 Figs).

Increasing stage of donor AKI was associated with DGF (stage 1 OR 1.85, 95% CI 1.50–2.28; stage 2 OR 2.24, 95% CI 1.61–3.11, stage 3 OR 4.77, 95% CI 3.25–7.01), but not with acute rejection (S3 Table).

## Discussion

This large Australia and New Zealand study of 10,101 kidney transplant recipients followed over a 21-year period demonstrated that donor AKI was not associated with an increased risk of all-cause graft failure after adjustment for donor, recipient and transplant characteristics. Similar results were observed for the secondary outcomes including death-censored graft failure, graft failure with death as a competing event and all-cause mortality. Donor AKI was not associated with acute rejection but was associated with an increased risk of DGF. Donor AKI stage was not associated with any kidney transplant outcome, except DGF. During the study period, the proportion of donors with AKI remained comparable, but a greater proportion of kidneys from donors with stage 3 AKI were transplanted in the most recent era (2010–2017), with comparable post-transplant outcomes.

The main finding of no significant association between donor AKI and all-cause graft failure is in keeping with that of a recent US Organ Procurement and Transplantation Network (OPTN) registry study [5], that also reported no association between donor AKI status and either all-cause graft survival or death-censored graft failure in 2,430 kidney transplant recipients between 2010 and 2013. This US study pointed out that the transplant community should consider measures to increase utilization of kidneys from deceased donors with AKI, especially in the context of organ shortage. Kayler et al. [12] analyzed 82,262 kidney transplant recipients recorded in the US Scientific Registry of Transplant Recipients between 1995 and 2007 and also found no significant association between donor AKI status and graft survival in recipients of standard criteria donor (SCD) kidneys. Similarly, Domagala et al. [11] reported a lack of association between graft survival and the presence of donor AKI in 226 kidney transplant recipients and Heilman et al. [21] analyzed 204 kidney transplant recipients at a single US centre between 2004 and 2013, and found no association between donor AKI status and graft survival in kidney transplant recipients.

In contrast, a previously published UK Transplant Registry of 11,219 deceased donor kidney transplant recipients between 2003 and 2013 reported that recipients of kidneys from donors with AKI were at increased risk of graft failure after adjustment for confounding factors, and experienced slightly inferior 1-, 3-, and 5-year graft survival rates compared with those who received kidneys from donors without AKI [7]. Although the difference was statistically significant, the absolute difference in graft survival between the two groups at each time point was only 2% (1-year transplant survival 89% vs. 91%; 3-year transplant survival 83% vs. 85%; 5-year transplant survival 76% vs. 78%). Nevertheless, the authors still concluded that while kidneys from donors with AKI stages 1 and 2 should be used, caution was advised for kidneys from donors with stage 3 AKI [7]. However, it should be noted that the proportion of recipients of kidneys from donors with AKI in this study was higher than in the current study (17% vs. 12%). In addition, the outcome of graft failure (defined similarly to the current study) was analyzed at exactly 1-year post transplant (rather than over the entire follow-up duration in the present study) and fewer variables were used for adjustment in multivariable modelling (8 versus 23 in the present study). These differences may have explained the apparent differences in findings between the studies. Schütte-Nütgen et al. [22] analyzed 214 kidney transplant recipients at a single German transplant center over 10 years and also reported that death-censored and overall graft survival were significantly lower for recipients receiving kidneys from donors with AKI compared to recipients without AKI. However, this study was limited by its small sample size and only performed univariable Cox regressions to examine the association of donor AKI with graft failure.

The current study also reported that all-cause mortality was comparable among recipients who received kidneys from donors with AKI or without AKI after full adjustment, and was not associated with the severity of AKI stage. Several small studies also reported a similar relationship with donor AKI and patient survival. Ali et al. [23] analyzed 284 kidney transplant recipients over 12 years and showed that donor AKI was not associated with patient survival. Schütte-Nütgen et al. [22] also found that donor AKI was not associated with transplant recipient survival, with estimated 5-year patient survival being similar between the two groups (92% and 86%, respectively) and noticeably higher than that of patients on the transplant waiting list (41%).

In the present study, the incidence of DGF was significantly higher in donors with AKI and the risk increased progressively with increasing AKI stage. This finding is consistent with that of previous studies [21, 24, 25]. DGF has in turn been identified as a risk factor for acute rejection and poorer long-term patient and graft outcomes [18, 26–28]. However, in this study, donor AKI was not associated with a higher rate of acute rejection. Our study confirmed that while kidneys with AKI are prone to develop DGF, long-term results comparable to those from donors without AKI can be achieved.

Another novel finding of the present study was that graft failure was not consistently associated with the severity of AKI, as defined by KDIGO stage. This may have been due in part to the relatively small numbers of events in recipients of kidneys with stage 2 and 3 AKI. Moreover, donor kidneys with stage 3 AKI were commonly utilized in the more recent era (2010–2017), and this era was associated with a lower risk of graft failure as compared to the earlier era. However, the shorter follow-up duration may have limited the ability to detect a difference in graft outcomes. There may have also been a component of selection bias, since kidneys with more severe AKI were more likely to have procurement biopsies and may have been discarded more often unless there were other favorable characteristics that supported their preferential selection for transplantation. For example, in the present study, donors with stage 3 AKI tended to be younger, were less likely to be ECD, and were more likely to have died from anoxia. Nevertheless, this result still encourages broader utilization of AKI stage 3 kidneys for transplantation.

A recent single center report on 1,113 deceased donor kidney transplants followed for 10 years from Mayo clinic investigators [25] showed no significant difference in all-cause graft failure between recipients with and without AKI. However, preimplantation biopsies were done in donors with AKI and only kidneys with < 10% cortical necrosis and no more than mild chronic changes in the interstitium, vessels or glomeruli were used for transplantation. Our study in comparison had a biopsy rate of only 28% for kidneys with AKI. While the Mayo Clinic policy promoting obligatory biopsies for all donor kidneys with AKI does have merit [29], it needs to be balanced against the potential for this practice to delay transplant procedures and lengthen ischemic times. Our current study showed that using kidneys from donors with AKI was associated with acceptable long-term graft and patient survival rates that were almost as good as those of recipients of donor kidneys without AKI. This suggests that our current transplant strategies are appropriate and acceptable.

In native kidneys, AKI is often associated with increased risks of chronic kidney disease (CKD) progression and ESKD, which increases in a graded manner with the severity of AKI [6, 30]. Potential mechanisms underpinning this progression of AKI to CKD include hypoxia, failed tubule differentiation, inflammation, vascular injury, endothelial cell dysfunction, interstitial fibrosis and α Klotho deficiency [31–34]. In the present study, AKI status and severity was associated with DGF, which may indirectly suggest reduced kidney function recovery in organs from donors with AKI, but this was not associated with an increase in the risk of long-term outcomes after full adjustment for confounders, such as graft failure and death. It seems reasonable to assume that these factors may operate differently in kidney transplantation and it is possible that the interplay of additional transplant-specific factors may impact renal clinical outcomes.

The strengths of this study lie in its large size and extended duration of follow up. It included all patients who received a deceased donor primary kidney-only transplant in Australia and New Zealand between 1997 and 2017, making the study population representative of a broad range of patient demographics and comorbidities. However, there were some limitations to this study. Firstly, the possibility of selection bias, particularly in donors with more severe AKI, could not be excluded. Even though adjustment was made for an appreciable number of donor, recipient and transplant characteristics, the possibility of residual confounding remained. Secondly, some donors already had significantly elevated SCr concentrations on admission without data on previous kidney function, thereby impairing the ability to diagnose donor AKI based on KDIGO criteria because of delayed and restricted time windows for assessment. Some degree of misclassification was therefore possible. Thirdly, the ANZOD Registry does not record information on duration of decreased urine output, which is one of the important criteria used by KDIGO to define AKI. The registry also does not collect other important information, such as procurement kidney biopsy results, kidney transplant management protocols and concomitant medications. Fourthly, there were relatively small numbers of donors with severe stage 3 AKI, which limited the ability to evaluate the association between donor AKI severity and transplant outcomes. Finally, AKI was solely defined according to the KDIGO AKI classification, which has been endorsed by both the Acute Dialysis Quality Initiative (ADQI) and KDIGO working groups [14, 35]. Use of alternative AKI classification systems, such as the Acute Kidney Injury Network (AKIN) or Risk, Injury, and Failure, and Loss, and End-stage kidney disease (RIFLE) definitions, may have led to different findings.

In conclusion, donor AKI was not associated with a significantly increased risk of all-cause graft failure, mortality, death-censored graft failure and graft failure with death as a competing risk after adjustment for donor, recipient and transplant characteristics. Thus, cautious use of donor kidneys with AKI appears justifiable in a number of kidney transplant circumstances, particularly if associated with immunologic advantage. Further studies are warranted to determine optimal selection and risk stratification of donor kidneys with AKI to help better inform shared decision-making regarding the anticipated benefits and risks of their use in specific recipients.

## Supporting information

S1 Fig Cumulative incidence of graft failure analysed with death as a competing event according to acute kidney injury status in first kidney transplant recipients in Australia and New Zealand 1997–2017 (sHR 1.07, 95% CI 0.92–1.24). (PNG)

S2 Fig Unadjusted Kaplan-Meier survival curve for all-cause graft survival according to donor acute kidney injury stage in first kidney transplant recipients in Australia and New Zealand 1997–2017 (p = 0.05). (PNG)

S3 Fig Unadjusted Kaplan-Meier survival curve for patient survival according to donor acute kidney injury stage in first kidney transplant recipients in Australia and New Zealand 1997–2017 (p = 0.08). (PNG)

S4 Fig Unadjusted Kaplan-Meier survival curve for death-censored graft survival according to donor acute kidney injury stage in first kidney transplant recipients in Australia and New Zealand 1997–2017 (p = 0.58). (PNG)

S5 Fig Cumulative incidence plot for death with functioning graft as a competing risk according to acute kidney injury stage in first kidney transplant recipients in Australia and New Zealand 1997–2017 (p = 0.81). (PNG)

S1 Table Donor characteristics according to AKI stage. (DOCX)

S2 Table Univariable analyses of exposure factors related to all-cause graft failure, death-censored graft failure, all-cause mortality and graft failure with death as a competing event. (DOCX)

S3 Table Multivariable logistic regression analyses of risks of delayed graft function and first episode of acute rejection within 6 months according to donor AKI status and stage in kidney transplant recipients in Australia and New Zealand 1997–2017. (DOCX)

S4 Table Associations between donor AKI stage with all cause graft failure, death-censored graft failure, all-cause mortality and graft failure with death as a competing event. (DOCX)

## References

[1] Wolfe RA, Ashby VB, L.Milford E, Ojo A o., E.Ettenger R, Y.C.Agodoa L, et al. Comparison of mortality in all patients on dialysis, patients on dialysis awaiting transplantation, and recipients of a first cadaveric transplant. N Engl J Med. 1999;341(23):1725–30. 10.1056/NEJM199912023412303 10580071

[2] Gill JS, Tonelli M, Johnson N, Kiberd B, Landsberg D, Pereira BJG, et al. The impact of waiting time and comorbid conditions on the survival benefit of kidney transplantation. Kidney Int. 2005;68(5):2345–51. 10.1111/j.1523-1755.2005.00696.x 16221239

[3] Merion RM, Ashby VB, Wolfe RA, DA D, TE H-S, RA M, et al. Deceased-donor characteristics and the survival benefit of kidney transplantation. J Am Med Assoc. 2005;294(21):2726–33. 10.1001/jama.294.21.2726 16333008

[4] Maggiore U, Oberbauer R, Pascual J, Viklicky O, Dudley C, Budde K, et al. Strategies to increase the donor pool and access to kidney transplantation: An international perspective. Nephrol Dial Transplant. 2015;30(2):217–22. 10.1093/ndt/gfu212 24907023 PMC4309190

[5] Hall IE, Akalin E, Bromberg JS, Doshi MD, Greene T, Harhay MN, et al. Deceased-donor acute kidney injury is not associated with kidney allograft failure. Kidney Int. 2019 1;95(1):199–209. 10.1016/j.kint.2018.08.047 30470437 PMC6331055

[6] Coca SG, Singanamala S, Parikh CR. Chronic kidney disease after acute kidney injury: a systematic review and meta-analysis. Kidney Int. 2012;81(5):442–8. 10.1038/ki.2011.379 22113526 PMC3788581

[7] Boffa C, van de Leemkolk F, Curnow E, Homan van der Heide J, Gilbert J, Sharples E, et al. Transplantation of Kidneys From Donors With Acute Kidney Injury: Friend or Foe? Am J Transplant. 2017;17(2):411–9. 10.1111/ajt.13966 27428556

[8] Hall IE, Schröppel B, Doshi MD, Ficek J, Weng FL, Hasz RD, et al. Associations of deceased donor kidney injury with kidney discard and function after transplantation. Am J Transplant. 2015;15(6):1623–31. 10.1111/ajt.13144 25762442 PMC4563988

[9] Kim JH, Kim YS, Choi MS, Kim YO, Yoon SA, Kim J Il, et al. Prediction of clinical outcomes after kidney transplantation from deceased donors with acute kidney injury: A comparison of the KDIGO and AKIN criteria. BMC Nephrol. 2017;18(1):1–13. 10.1186/s12882-016-0417-1 28129763 PMC5273789

[10] Jung CW, Park KT, Kim SY, Kim SJ, Kim MG, Jo SK, et al. Clinical outcomes in kidney transplantation patients from deceased donors with acute kidney injury. Transplant Proc. 2013;45(8):2941–5. 10.1016/j.transproceed.2013.08.048 24157008

[11] Domagala P, Gorski L, Wszola M, Kieszek R, Diuwe P, Goralski P, et al. Successful transplantation of kidneys from deceased donors with terminal acute kidney injury. Ren Fail. 2019;41(1):167–74. 10.1080/0886022X.2019.1590209 30909784 PMC6442227

[12] Kayler LK, Howard R, Schold JD. Outcomes and Utilization of Kidneys from Deceased Donors with Acute Kidney Injury. Am J Transplant. 2009;9:367–73. 10.1111/j.1600-6143.2008.02505.x 19178415

[13] Elm E von Altman DG, Egger M Pocock SJ, Gøtzsche PC Vandenbroucke JP, et al. The strengthening the reporting of observational studies in epidemiology (STROBE) statement: Guidelines for reporting observational studies. J Clin Epidemiol. 2008;61(12):344–9. 18313558 10.1016/j.jclinepi.2007.11.008

[14] KDIGO AKI Work Group. KDIGO clinical practice guideline for acute kidney injury. Kidney Int Suppl. 2012;2(1):1–138.

[15] Stratta RJ, Rohr MS, Sundberg AK, Armstrong G, Hairston G, Hartmann E, et al. Increased Kidney Transplantation Utilizing Expanded Criteria Deceased Organ Donors with Results Comparable to Standard Criteria Donor Transplant. 2004;239(5):688–97. 10.1097/01.sla.0000124296.46712.67 PMC1356277 15082973

[16] Rao PS, Schaubel DE, Guidinger MK, Andreoni KA, Wolfe RA, Merion RM, et al. A Comprehensive Risk Quantification Score for Deceased Donor Kidneys: The Kidney Donor Risk Index. Transplantation. 2009;88(2):231–6. 10.1097/TP.0b013e3181ac620b 19623019

[17] Israni AK, Salkowski N, Gustafson S, Snyder JJ, Friedewald JJ, Formica RN, et al. New national allocation policy for deceased donor kidneys in the United States and possible effect on patient outcomes. J Am Soc Nephrol. 2014;25(8):1842–8. 10.1681/ASN.2013070784 24833128 PMC4116061

[18] Levey AS, Coresh J, Greene T, Stevens LA, Zhang Y (Lucy), Hendriksen S, et al. Using Standardized Serum Creatinine Values in the Modification of Diet in Renal Disease Study Equation for Estimating Glomerular Filtration Rate. Ann Intern Med. 2006;145(4):247–55. 10.7326/0003-4819-145-4-200608150-00004 16908915

[19] Fine JP, Gray RJ. A Proportional Hazards Model for the Subdistribution of a Competing Risk. J Am Stat Assoc. 1999;94(446):496–509.

[20] Zamir O, Etzioni O. Web Document Clustering: A Feasibility Demonstration. Proceedings of the 21th international ACM SIGIR conference on Research and Development in information Retrival (SIGIR’98). 1998; pp 46–54.

[21] Heilman RL, Smith ML, Kurian SM, Huskey J, Batra RK, Chakkera HA, et al. Transplanting Kidneys From Deceased Donors With Severe Acute Kidney Injury. 2015;2143–51. 10.1111/ajt.13260 25808278

[22] Schütte-Nütgen K, Finke M, Ehlert S, Thölking G, Pavenstädt H, Suwelack B, et al. Expanding the donor pool in kidney transplantation: Should organs with acute kidney injury be accepted?—A retrospective study. PLoS One. 2019;14(3):1–14. 10.1371/journal.pone.0213608 30865677 PMC6415810

[23] Ali T, Dimassi W, Elgamal H, Alabassi A, Aleid H, Altalhi M, et al. Outcomes of kidneys utilized from deceased donors with severe acute kidney injury. QJMed. 2015;108(10):803–11. 10.1093/qjmed/hcv033 25660604

[24] Lee MH, Jeong E, Chang JY, Kim Y, Kim J, Moon IS, et al. Clinical outcome of kidney transplantation from deceased donors with acute kidney injury by Acute Kidney Injury Network criteria. J Crit Care. 2014;29(3):432–7. 10.1016/j.jcrc.2013.12.016 24468572

[25] Heilman RL, Smith ML, Smith BH, Kumar A, Srinivasan A, Huskey JL, et al. Long-term outcomes following kidney transplantation from donors with acute kidney injury. Transplantation. 2019;103(9):1. 10.1097/TP.0000000000002792 31205261

[26] Pieringer H, Biesenbach G. Risk factors for delayed kidney function and impact of delayed function on patient and graft survival in adult graft recipients. clin Transpl. 2005;19(3):391–8. 10.1111/j.1399-0012.2005.00360.x 15877804

[27] Troppmann C, Gillingham KJ, Benedetti E, Almond PS, Rainer W.Gruessner G., Najarian JS, et al. Delayed graft function, acute rejection, and outcome after cadaver renal transplantation. The multivariate analysis. Transplantation. 1995;59(7):962–8. 10.1097/00007890-199504150-00007 7709456

[28] Lim WH, Johnson DW, Teixeira-pinto A, Wong G. Association Between Duration of Delayed Graft Function, Acute Rejection, and Allograft Outcome After Deceased Donor Kidney Transplantation. Transplantation. 2019;103(2):412–9. 10.1097/TP.0000000000002275 29762458

[29] Randhawa PS. Role of preimplantation biopsies in kidney donors with acute kidney injury. Transplantation. 2019;103(9):1. 10.1097/TP.0000000000002791 31205258

[30] See EJ, Jayasinghe K, Glassford N, Bailey M, Johnson DW, Polkinghorne KR, et al. Long-term risk of adverse outcomes after acute kidney injury: a systematic review and meta-analysis of cohort studies using consensus definitions of exposure. Kidney Int. 2019 1;95(1):160–72. 10.1016/j.kint.2018.08.036 30473140

[31] Tanaka S, Tanaka T, Nangaku M. Hypoxia as a key player in the AKI-to-CKD transition. Am J Physiol—Ren Physiol. 2014;307(11):F1187–95. 10.1152/ajprenal.00425.2014 25350978

[32] Shi M, Flores B, Gillings N, Bian A, Cho HJ, Yan S, et al. Αklotho Mitigates Progression of Aki To Ckd Through Activation of Autophagy. J Am Soc Nephrol. 2016;27(8):2331–45. 10.1681/ASN.2015060613 26701976 PMC4978045

[33] Takaori K, Yanagita M. Insights into the Mechanisms of the Acute Kidney Injury-to-Chronic Kidney Disease Continuum. Nephron. 2016;134(3):172–6. 10.1159/000448081 27398799

[34] Venkatachalam MA, Weinberg JM, Kriz W, Bidani AK. Failed tubule recovery, AKI-CKD transition, and kidney disease progression. J Am Soc Nephrol. 2015;26(8):1765–76. 10.1681/ASN.2015010006 25810494 PMC4520181

[35] Chawla LS, Bellomo R, Bihorac A, Goldstein SL, Siew ED, Bagshaw SM, et al. Acute kidney disease and renal recovery: Consensus report of the Acute Disease Quality Initiative (ADQI) 16 Workgroup. Nat Rev Nephrol. 2017;13(4):241–57. 10.1038/nrneph.2017.2 28239173

