# Supplementary figures and images for: Impact of deceased donor with acute kidney injury on subsequent kidney transplant outcomes–an ANZDATA registry analysis
Source: PLoS One. 2021 Mar 25;16(3):e0249000. doi: 10.1371/journal.pone.0249000 (PMC7993825; doi:10.1371/journal.pone.0249000)

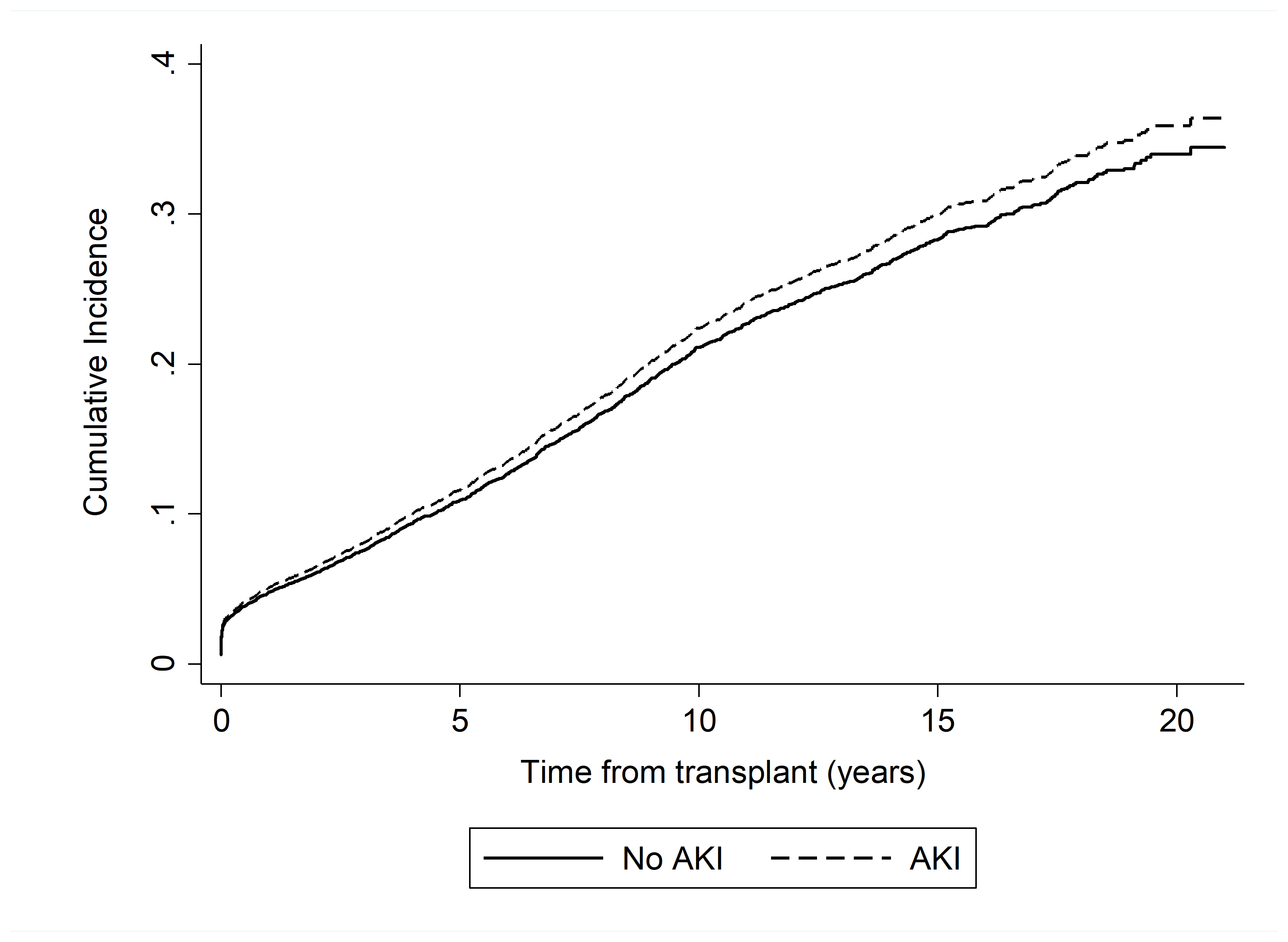

Supplement: S1 Fig — (PNG) [file pone.0249000.s001.png]

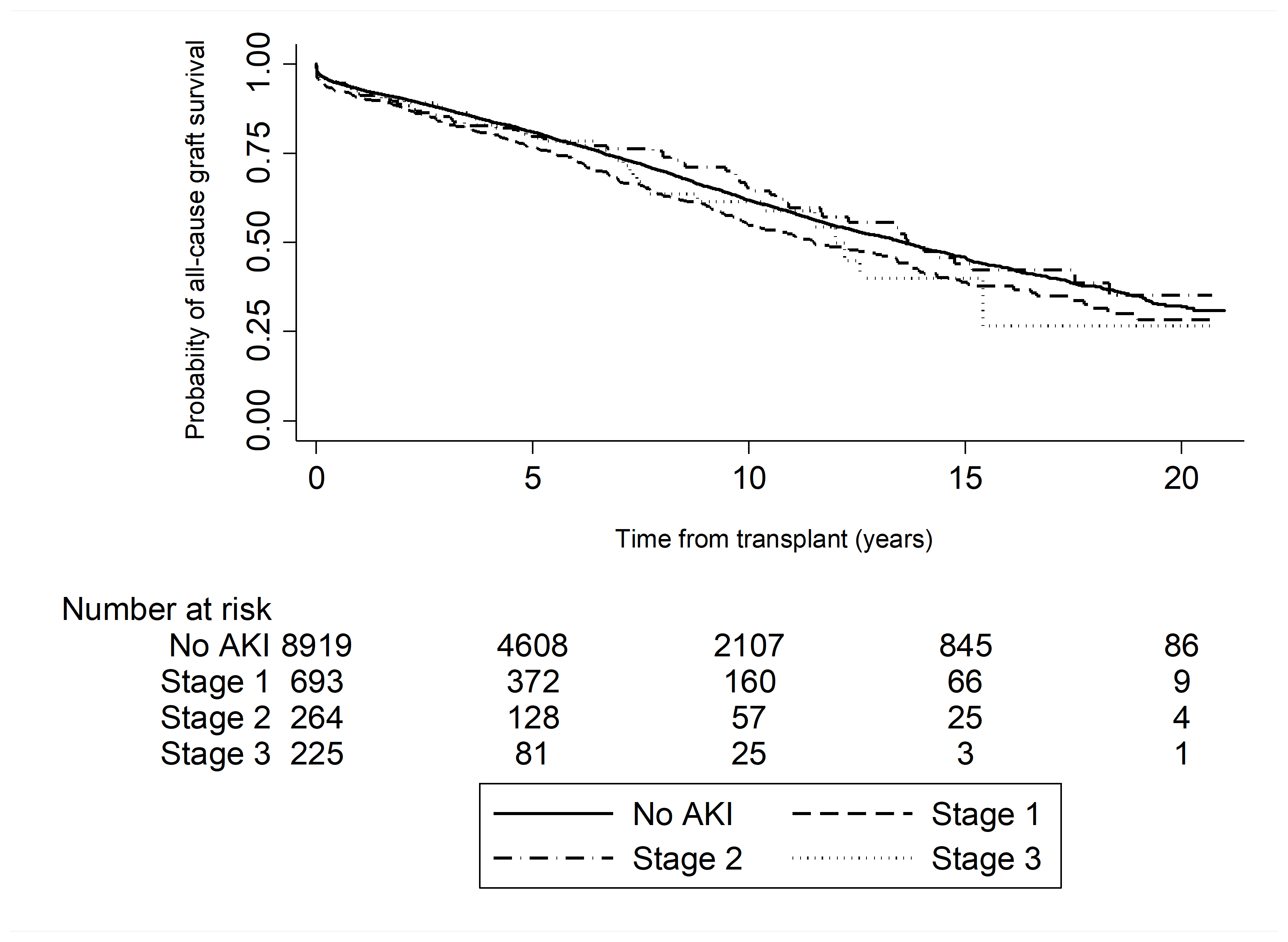

Supplement: S2 Fig — (PNG) [file pone.0249000.s002.png]

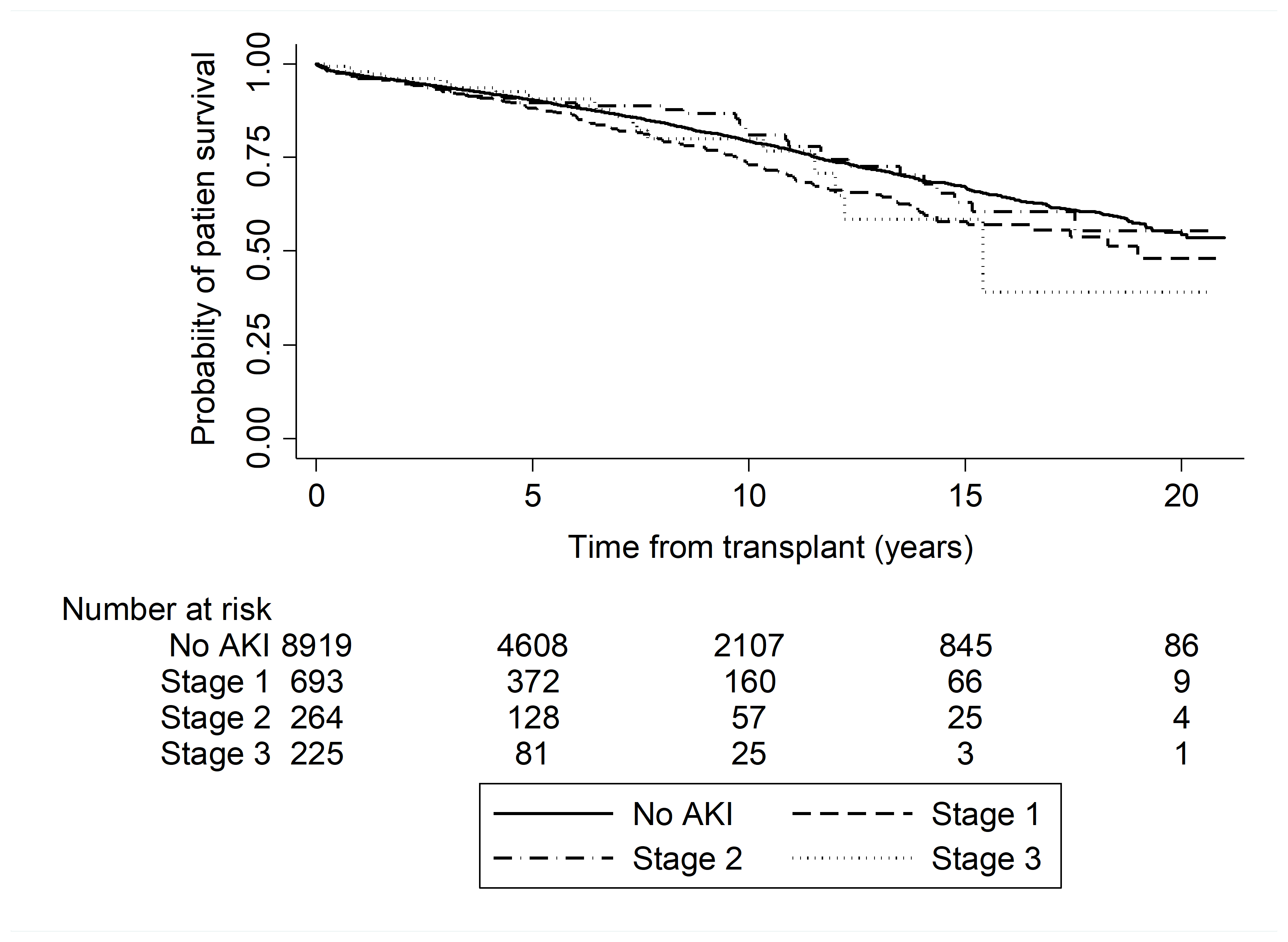

Supplement: S3 Fig — (PNG) [file pone.0249000.s003.png]

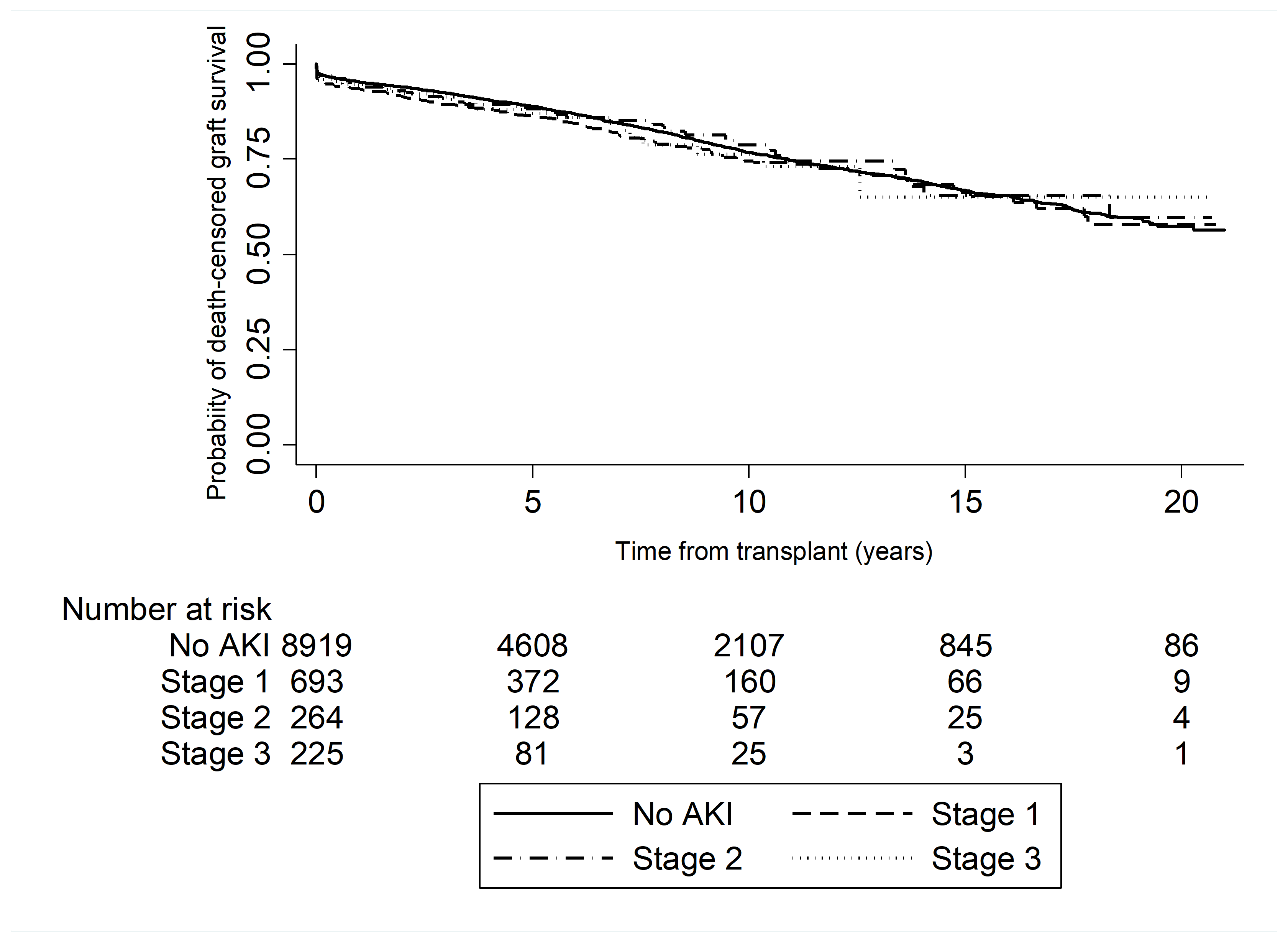

Supplement: S4 Fig — (PNG) [file pone.0249000.s004.png]

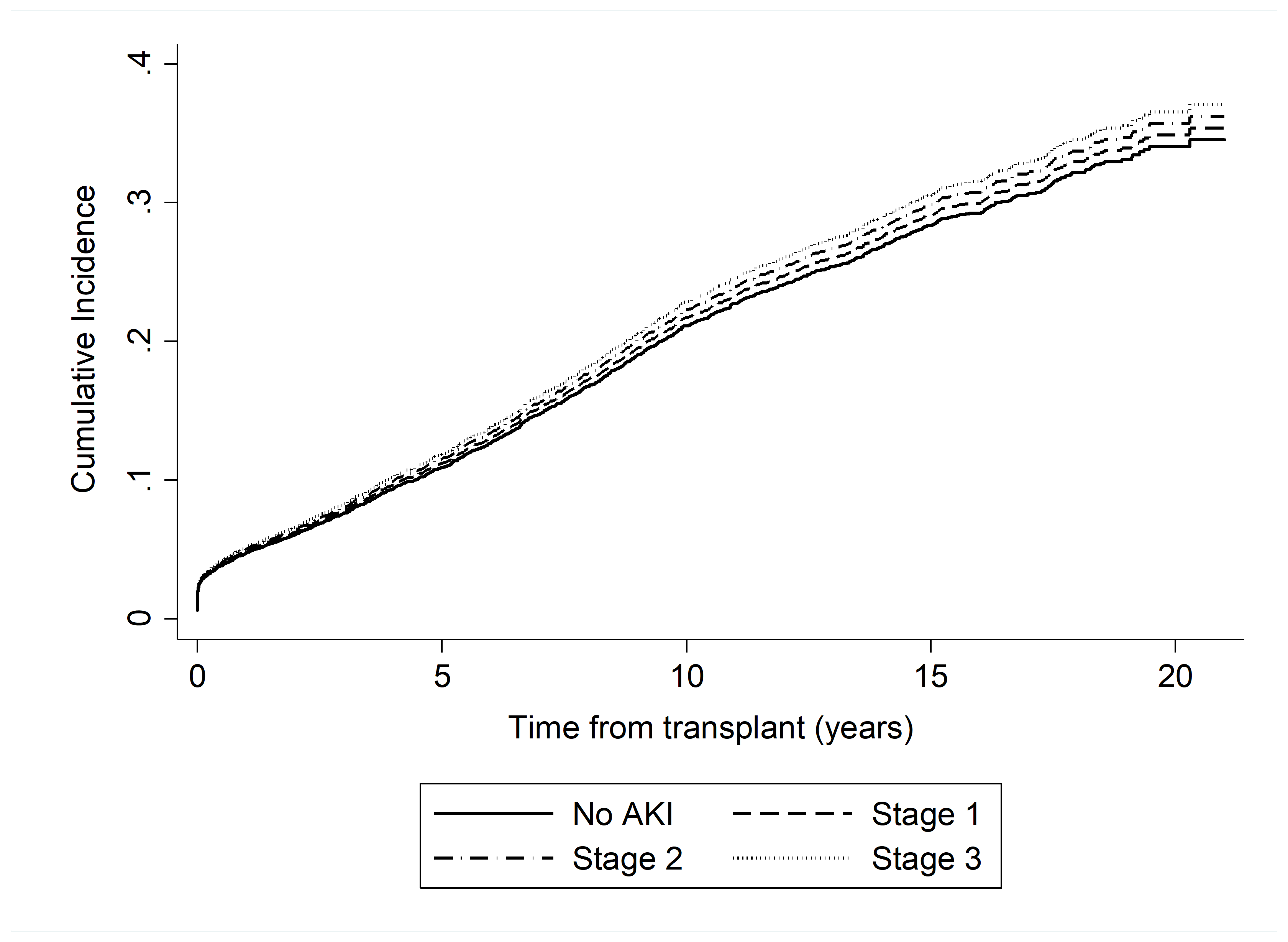

Supplement: S5 Fig — (PNG) [file pone.0249000.s005.png]
