# Supplementary material for: Impact of deceased donor with acute kidney injury on subsequent kidney transplant outcomes–an ANZDATA registry analysis
Source: PLoS One. 2021 Mar 25;16(3):e0249000. doi: 10.1371/journal.pone.0249000 (PMC7993825; doi:10.1371/journal.pone.0249000)
Supplement: S1 Table — (DOCX) [file pone.0249000.s006.docx]

**S1 Table. Donor characteristics according to AKI stage.**

| **Donor characteristics** | **All AKI**  **(N=662)** | **AKI stage 1**  **(N=393)** | **AKI stage 2**  **(N=143)** | **AKI stage 3**  **(N=126)** | **P value** |
| --- | --- | --- | --- | --- | --- |
| Age | 46 (30, 58) | 50 (31, 60) | 46 (32, 57) | 42 (27, 47) | <0.01 |
| Male | 410 (62%) | 239 (61%) | 95 (66%) | 76 (60%) | 0.46 |
| Ethnicity^#^ | | | | | 0.10 |
| Caucasoid | 611 (92%) | 367 (93%) | 132 (92%) | 112 (89%) |  |
| Asian | 31(5%) | 18 (5%) | 8 (6%) | 5 (4%） |  |
| Māori | 6 (<1%) | 2 (<1%) | 0 (<1%) | 4 (3%) |  |
| Island | 3 (<1%) | 1 (<1%) | 0 (<1%) | 2 (2%) |  |
| Other | 11 (2%) | 5 (1%) | 3 (2%) | 3 (2%) |  |
| Weight, kg | 80 (68, 90) | 80 (67, 90) | 80 (70, 90) | 80 (70, 97) | 0.21 |
| Height, cm | 172 (165, 180) | 172 (164, 180) | 173 (166, 180) | 172 (163, 180) | 0.43 |
| Diabetes | 43 (7%) | 25 (6%) | 8 (6%) | 10 (8%) | 0.74 |
| Hypertension | 149 (23%) | 90 (23%) | 41 (29%) | 18 (15%) | 0.02 |
| Smoking^#^ |  |  |  |  | 0.09 |
| Current | 254 (38%) | 146 (37%) | 65 (46%) | 43 (34%) |  |
| Former | 124 (19%) | 74 (19%) | 31 (22%) | 19 (15%) |  |
| Never | 273 (41%) | 165 (42%) | 45 (32%) | 63 (50%) |  |
| Unknown | 11 (2%) | 8 (2%) | 2 (1%) | 1 (<1%) |  |
| Cause of death^#^ | | | | | <0.01 |
| Head trauma | 130 (20%) | 79 (20%) | 34 (24%) | 17 (14%) |  |
| Anoxia | 232 (35%) | 103 (26%) | 47 (33%) | 82 (65%) |  |
| Cerebrovascular/stroke | 260 (39%) | 183 (47%) | 59 (42%) | 18 (14%) |  |
| CNS tumor | 3 (<1%) | 3 (<1%) | 0 (<1%) | 0 (<1%) |  |
| Other | 37 (6%) | 25 (6%) | 3 (2%) | 9 (7%) |  |
| DCD | 99 (15%) | 58 (15%) | 18 (13%) | 23 (18%) | 0.43 |
| Hepatitis C seropositive^#^ | 1 (<1%)) | 0 (<1%) | 0 (<1%) | 1 (<1%) | 0.19 |
| ECD | 208 (32%) | 142 (36%) | 48 (34%) | 18 (14%) | <0.01 |
| KDRI | 1.3 (1.1, 1.7) | 1.4 (1.1, 1.8) | 1.4 (1.1, 1.8) | 1.3 (1.1, 1.5) | 0.03 |
| KDPI, % | 62 (38, 84) | 69 (38, 86) | 64 (39, 85) | 51 (37, 68) | <0.01 |
| Admission to procurement, days | 2.1 (1.5, 3.8) | 1.9 (1.4, 3.5) | 2.3 (1.6, 4.3) | 2.9 (2.0, 4.2) | <0.01 |
| Admission SCr, µmol/L | 85 (64, 111) | 80 (60, 108) | 84 (62, 103) | 102 (76, 145) | <0.01 |
| Terminal SCr, µmol/L | 163 (110, 250) | 129 (98, 170) | 181 (150, 241) | 401 (283, 476) | <0.01 |
| Terminal urine output, ml/kg/h | 0.9 (0.5, 1.5) | 1 (0.6, 1.8) | 0.7 (0.5, 1.4) | 0.6 (0.3, 1.0) | <0.01 |
| Oliguria last 12 hours (<20mls/h) | 148 (22%) | 73 (19%) | 36 (25%) | 39 (31%) | 0.01 |
| Oliguria duration, hours | 3 (1, 7) | 2 (1, 4) | 4 (1.5, 6.5) | 7 (2, 12) | <0.01 |
| Procurement biopsy performed | 167(28%) | 84(24%) | 29(23%) | 54(44%) | <0.01 |
| Number of individual kidneys transplanted | | | | | 0.62 |
| 1 | 51 (8%) | 30 (8%) | 9 (6%) | 12 (10%) |  |
| 2 | 611 (92%) | 363 (92%) | 134 (94%) | 114 (90%) |  |
| Era |  |  |  |  | <0.01 |
| 1997-2003 | 157 (24%) | 109 (28%) | 36 (25%) | 12 (10%) |  |
| 2004-2010 | 192 (29%) | 123 (31%) | 38 (27%) | 31 (25%) |  |
| 2011-2017 | 313 (47%) | 161 (41%) | 69 (48%) | 83 (66%) |  |

Results are presented as medians (interquartile range) or frequency (percentage). Kruskal-Wallis test for continuous variables. Chi-square or Fisher’s exact test for categorical variables.

Abbreviations: AKI, acute kidney injury; DCD, donation after cardiovascular determination of death; ECD, expanded-criteria donor; KDPI, kidney donor profile index; KDRI, kidney donor risk index; SCr, serum creatinine; CNS, central nervous system.

#Using Fisher’s exact test.
