# Supplementary material for: Impact of deceased donor with acute kidney injury on subsequent kidney transplant outcomes–an ANZDATA registry analysis
Source: PLoS One. 2021 Mar 25;16(3):e0249000. doi: 10.1371/journal.pone.0249000 (PMC7993825; doi:10.1371/journal.pone.0249000)
Supplement: S2 Table — (DOCX) [file pone.0249000.s007.docx]

**S2 Table. Univariable analyses of exposure factors related to all-cause graft failure, death-censored graft failure, all-cause mortality and graft failure with death as a competing event.**

| **Factors** | **All-cause graft failure*** | | **Death-censored graft failure*** | | **Graft failure with death as a competing event^#^** | | **All-cause mortality*** | |
| --- | --- | --- | --- | --- | --- | --- | --- | --- |
|  |  |  |  |  |  |  |  |  |
|  | **HR (95% CI)** | **P value** | **HR (95% CI)** | **P value** | **SHR (95% CI)** | **P value** | **HR (95% CI)** | **P value** |
|  |  |  |  |  |  |  |  |  |
| Donor characteristics | | | | | | | | |
| AKI group | |  |  |  |  |  |  |  |
| No AKI | Ref |  | Ref |  | Ref |  | Ref |  |
| AKI | 1.14 (1.01-1.28) | 0.03 | 1.10 (0.94-1.28) | 0.24 | 1.07 (0.92-1.24) | 0.39 | 1.17 (1.01- 1.37) | 0.04 |
| KDPI components |  |  |  |  |  |  |  |  |
| Age | 1.01 (1.01-1.02) | <0.01 | 1.02 (1.01-1.02) | <0.01 | 1.01 (1.01-1.02) | <0.01 | 1.01 (1.01-1.01) | <0.01 |
| Height | 0.99 (0.99-0.99) | <0.01 | 0.99 (0.99-0.99) | <0.01 | 0.99 (0.99-0.99) | <0.01 | 1.00 (0.99-1.00) | 0.09 |
| Weight | 1.00 (0.99-1.00) | 0.54 | 1.00 (0.99-1.00) | 0.39 | 1.00 (0.99-1.00) | 0.15 | 1.00 (0.99-1.00) | 0.09 |
| Ethnicity |  | 0.09 |  | 0.56 |  | 0.65 |  | 0.31 |
| Caucasoid | Ref |  | Ref |  | Ref |  | Ref |  |
| Aboriginal/Torres Str | 0.89 (0.59-1.34) |  | 0.87 (0.52-1.46) |  | 0.84 (0.49-1.42) |  | 0.95 (0.51-1.77) |  |
| Asian | 1.11 (0.93-1.33) |  | 1.15 (0.91-1.45) |  | 1.12 (0.89-1.42) |  | 1.12 (0.87-1.45) |  |
| Māori | 1.20 (0.86-1.69) |  | 1.16 (0.76-1.78) |  | 1.11 (0.73-1.68) |  | 1.24(0.79-1.94) |  |
| Islander | 0.55 (0.29-1.04) |  | 0.72 (0.31-1.66) |  | 0.76 (0.33-1.75) |  | 0.49 (0.18-1.36) |  |
| Other | 0.68 (0.45-1.04) |  | 0.70 (0.38-1.31) |  | 0.70 (0.37-1.31) |  | 0.61 (0.32-1.15) |  |
| Hypertension | 1.51 (1.38-1.64) | <0.01 | 1.62 (1.45-1.82) | <0.01 | 1.52 (1.35-1.70) | <0.01 | 1.39 (1.23-1.56) | <0.01 |
| Diabetes | 1.26 (1.07-1.49) | <0.01 | 1.27 (1.01-1.59) | 0.04 | 1.21 (0.96-1.51) | 0.1 | 1.27 (1.02-1.58) | 0.03 |
| Cause of Death |  | <0.01 |  | <0.01 |  | <0.01 |  | <0.01 |
| Head truama | 0.71 (0.65-0.78) |  | 0.62 (0.55-0.70) |  | 0.65 (0.58-0.74) |  | 0.80 (0.71-0.90) |  |
| Anoxia | 0.74 (0.67-0.82) |  | 0.71 (0.62-0.82) |  | 0.71 (0.62-0.82) |  | 0.75 (0.65-0.87) |  |
| Cerebrovascular/stroke | Ref |  | Ref |  | Ref |  | Ref |  |
| CNS tumour | 0.72 (0.53-0.98) |  | 0.65 (0.43-0.97) |  | 0.69 (0.46-1.02) |  | 0.88 (0.58-1.33) |  |
| Other | 0.74 (0.62-0.88) |  | 0.63 (0.50-0.81) |  | 0.64 (0.50-0.81) |  | 0.82 (0.64-1.04) |  |
| Terminal SCr, µmol/L | 1.00 (0.99-1.00) | 0.2 | 1.00 (0.99-1.00) | 0.18 | 1.00 (0.99-1.00) | 0.22 | 1.00 (0.99-1.00) | 0.42 |
| HCV Status | 1.72 (1.08-2.75) | 0.02 | 1.16 (0.55-2.42) | 0.69 | 0.94 | 0.88 | 2.45 (1.39-4.30) | <0.01 |
| DCD | 0.73 (0.72-0.95) | <0.01 | 0.74 (0.61-0.90) | <0.01 | 0.69 (0.57-0.84) | <0.01 | 0.93 (0.76-1.12) | 0.44 |
| Other donor factors | | | | | | | | |
| Sex (Male) | 0.92 (0.86-0.99) | 0.02 | 0.86 (0.78-0.95) | <0.01 | 0.87 (0.79-0.96) | <0.01 | 0.98 (0.88-1.08) | 0.63 |
| Number of individual kidneys transplanted |  |  |  |  |  |  |  |  |
| 1 | 1.39 (1.15-1.70) | <0.01 | 1.51 (1.17-1.94) | <0.01 | 1.44 (1.12-1.86) | <0.01 | 1.28 (0.96-1.71) | 0.09 |
| 2 | Ref |  | Ref |  | Ref |  | Ref |  |
| Procurement biopsy performed | 1.48 (1.33-1.65) | <0.01 | 1.56 (1.34-1.80) | <0.01 | 1.50 (1.30-1.73) | <0.01 | 1.44 (1.25-1.67) | <0.01 |
| Recipient  characteristics | | |  | |  |  |  | |
| Age |  | <0.01 |  | <0.01 |  | <0.01 |  | <0.01 |
| <18yr | 1.05 (0.85-1.30) |  | 2.32 (1.86-2.89) |  | 2.62 (2.11-3.26) |  | 0.15 (0.08-0.30) |  |
| 18-29yr | 0.88 (0.76-1.02) |  | 1.99 (1.69-2.34) |  | 2.32 (1.97-2.72) |  | 0.13 (0.09-0.20) |  |
| 30-39yr | 0.85 (0.76-0.95) |  | 1.64 (1.42-1.89) |  | 1.85 (1.60-2.13) |  | 0.29 (0.24-0.36) |  |
| 40-49yr | 0.82 (0.74-0.90) |  | 1.22 (1.06-1.39) |  | 1.31 (1.14-1.50) |  | 0.53 (0.46-0.61) |  |
| 50-59yr | Ref |  | Ref |  | Ref |  | Ref |  |
| 60-69yr | 1.31 (1.19-1.44) |  | 0.91 (0.77-1.07) |  | 0.82 (0.70-0.97) |  | 1.64 (1.46-1.84) |  |
| >=70yr | 0.72 (1.41-2.10) |  | 0.95 (0.62-1.46) |  | 0.79(0.51-1.21) |  | 2.46 (1.96-3.09) |  |
| Sex (Male) | 1.12 (1.04-1.20) | <0.01 | 1.09 (0.99-1.20) | 0.09 | 1.07(0.97-1.18) | 0.19 | 1.15 (1.04-1.28) | <0.01 |
| Ethnicity origin |  | <0.01 |  | <0.01 |  | <0.01 |  |  |
| Caucasoid | Ref |  | Ref |  | Ref |  | Ref |  |
| Aboriginal/Torres Str | 2.09 (1.81-2.41) |  | 2.24 (1.87-2.69) |  | 1.94 (1.62-2.33) |  | 1.96 (1.69-2.41) |  |
| Asian | 0.87 (0.76-0.98) |  | 1.00 (0.86-1.18) |  | 1.02 (0.87-1.20) |  | 0.75 (0.62-0.90) |  |
| Māori | 1.69(1.39-2.05) |  | 1.97 (1.54-2.52) |  | 1.84 (1.44-2.35) |  | 1.52 (1.14-2.02) |  |
| Pacific | 1.51 (1.24-1.84) |  | 2.13 (1.70-2.67) |  | 2.11 (1.69-2.65) |  | 0.80 (0.55-1.18) |  |
| Other | 0.73 (0.54-1.00) |  | 0.96 (0.67-1.41) |  | 1.00 (0.70-1.15) |  | 0.42 (0.24-0.74) |  |
| BMI |  | <0.01 |  | <0.01 |  | <0.01 |  | <0.01 |
| <18.5 | 1.11 (0.91-1.36) |  | 1.11 (0.91-1.36) |  | 1.53 (1.21-1.93) |  | 0.67( 0.47-0.96) |  |
| 18.5-24.9 | Ref |  | Ref |  | Ref |  | Ref |  |
| 25-29.9 | 1.01 (0.92-1.09) |  | 0.94 |  | 0.93 (0.83-1.05) |  | 1.08 (0.95-1.22) |  |
| 30-39.9 | 1.15 (1.05-1.26) |  | 1.07 |  | 1.02 (0.89-1.16) |  | 1.22 (1.07-1.40) |  |
| >=40 | 1.29 (1.09-1.52) |  | 1.46 |  | 1.42 (1.16-1.75) |  | 1.17 (0.77-1.78) |  |
| Previous transplant | 1.26 (1.14-1.40) | <0.01 | 1.56 (1.37-1.78) | <0.01 | 1.58 (1.29-1.79) | <0.01 | 0.96 (0.82-1.13) | 0.63 |
| Pre-emptive transplant | 0.61 (0.38-0.98) | 0.04 | 0.57 (0.29-1.11) | 0.10 | 0.59(0.30-1.15) | 0.12 | 0.62 (0.30-1.25) | 0.18 |
| Cause of ESKD |  | <0.01 |  | <0.01 |  | <0.01 |  | <0.01 |
| Diabetes | 1.66 (1.49-1.84) |  | 1.22 (1.05-1.43) |  | 1.06 (0.91-1.23) |  | 2.27 (1.96-2.63) |  |
| Glomerulonephritis | Ref |  | Ref |  | Ref |  | Ref |  |
| Hypertension | 1.21 (1.04-1.41) |  | 0.76 (0.59-0.98) |  | 0.69 (0.54-0.89) |  | 1.89 (1.56-2.29) |  |
| Polycystic Disease | 0.83 (0.74-0.93) |  | 0.52 (0.43-0.62) |  | 0.51 (0.42-0.61) |  | 1.25 (1.08-1.45) |  |
| Reflux Nephropathy | 0.96 (0.84-1.09) |  | 1.14 (0.97-1.32) |  | 1.18 (1.01-1.38) |  | 0.70 (0.56-0.88) |  |
| Other | 1.26 (1.14-1.39) |  | 1.18 (1.03-1.34) |  | 1.12 (0.99-1.28) |  | 1.39 (1.21-1.61) |  |
| Dialysis duration | 1.00 (1.00-1.00) | <0.01 | 1.00 (1.00-1.00) | <0.01 | 1.00(1.00-1.00) | <0.01 | 1.00 (1.00-1.00) | <0.01 |
| Induction immunosuppression | | | | | | | | |
| T cell depletion | 1.39 (1.22-1.57) | <0.01 | 1.50 (1.27-1.77) | <0.01 | 1.48 (1.25-1.74) | <0.01 | 1.27 (1.06-1.52) | <0.01 |
| B cell depletion | 1.93 (1.04-3.59) | 0.04 | 1.91 (0.85-4.31) | 0.12 | 1.64 (0.72-3.74) | 0.24 | 1.82 (0.74-4.48) | 0.19 |
| HLA mismatch level | 1.06 (1.04-1.08) | <0.01 | 1.09 (1.06-1.12) | <0.01 | 1.08 (1.05-1.11) | <0.01 | 1.02 (0.99-1.05) | 0.12 |
| Peak PRA (%)* | 1.01 (1.00-1.01) | <0.01 | 1.01 (1.01-1.01) | <0.01 | 1.01 (1.01-1.01) | <0.01 | 1.00 (1.00-1.00) | <0.01 |
| Transplant characteristics | | | | | | | | |
| Total ischemia time | 1.02 (1.02-1.03) | <0.01 | 1.02 (1.02-1.03) | <0.01 | 1.02 (1.01-1.03) | <0.01 | 1.03 (1.02-1.04) | <0.01 |
| Era |  | <0.01 |  | <0.01 |  | <0.01 |  | 0.04 |
| 1997-2003 | Ref |  | Ref |  | Ref |  | Ref |  |
| 2004-2010 | 0.95 (0.87-1.03) |  | 0.85 (0.75-0.95) |  | 0.77 (0.69-0.86) |  | 1.02 (0.91-1.15) |  |
| 2010-2017 | 0.76 (0.68-0.85) |  | 0.64 (0.55-0.75) |  | 0.56 (0.49-0.65) |  | 0.84 (0.71-0.99) |  |

Data presented as HR with 95% confidence interval (95%Cl) from Cox regression models (^*^ for all-cause graft failure, death-censored graft failure and all-cause mortality) or as sub-distribution (sHR) with 95%CI from competing risk model (^#^ for graft loss with death as competing event).

Abbreviations: AKI, acute kidney injury; DCD, donation after cardiovascular determination of death; ECD, expanded-criteria donor; KDPI, kidney donor profile index; KDRI, kidney donor risk index; SCr, serum creatinine; ESKD, End stage kidney disease; HD, hemodialysis; PD, peritoneal dialysis; HLA, human leukocyte antigen; Ivig, intravenous immunoglobulin; PRA, panel reactive antibody;
