# Supplementary material for: Impact of deceased donor with acute kidney injury on subsequent kidney transplant outcomes–an ANZDATA registry analysis
Source: PLoS One. 2021 Mar 25;16(3):e0249000. doi: 10.1371/journal.pone.0249000 (PMC7993825; doi:10.1371/journal.pone.0249000)
Supplement: S3 Table — (DOCX) [file pone.0249000.s008.docx]

**S3 Table. Multivariable logistic regression analyses of risks of delayed graft function and first episode of acute rejection within 6 months according to donor AKI status and stage in kidney transplant recipients in Australia and New Zealand 1997-2017.**

| **Outcome** | **Failures** | **Odds ratio (95% Cl)** | **P value** |
| --- | --- | --- | --- |
| Delayed Graft Function | |  | |
| No AKI (n=8919) | 2275 | Ref. |  |
| AKI-overall |  | 2.27 (1.92- 2.68) | <0.01 |
| Stage 1 (n=693) | 271 | 1.85 (1.50- 2.28) | <0.01 |
| Stage 2 (n=264) | 115 | 2.24 (1.61- 3.11) | <0.01 |
| Stage 3 (n=225) | 137 | 4.77 (3.25- 7.01) | <0.01 |
| First episode of acute rejection | |  | |
| No AKI (n=8919) | 2016 | Ref. |  |
| AKI-overall |  | 1.06 (0.88- 1.28) | 0.55 |
| Stage 1 (n=693) | 150 | 1.05 (0.84- 1.32) | 0.65 |
| Stage 2 (n=264) | 44 | 0.82 (0.54- 1.26) | 0.36 |
| Stage 3 (n=225) | 49 | 1.43 (0.96- 2.14) | 0.08 |

Model adjusted for donor characteristics: KDRI components except terminal serum creatinine (age, height, weight, ethnicity, history of hypertension, diabetes, cause of death, hepatitis C seropositivity, and donation after circulatory death [DCD]), gender, number of individual kidneys transplanted. Recipient and transplant characteristics: age, gender, ethnicity, body mass index [BMI], previous transplant, pre-emptive transplant, cause of ESKD, dialysis vintage, anti-rejection immunosuppression, number of HLA-mismatches, peak panel reactive antibody, total ischemia time, era (1997-2003, 2004-2010, 2011-2017).

Abbreviations: AKI, acute kidney injury; Cl, confidence interval.
