# Supplementary material for: Impact of deceased donor with acute kidney injury on subsequent kidney transplant outcomes–an ANZDATA registry analysis
Source: PLoS One. 2021 Mar 25;16(3):e0249000. doi: 10.1371/journal.pone.0249000 (PMC7993825; doi:10.1371/journal.pone.0249000)
Supplement: S4 Table — (DOCX) [file pone.0249000.s009.docx]

**S4 Table. Associations between donor AKI stage with all cause graft failure, death-censored graft failure, all-cause mortality and graft failure with death as a competing event.**

| **Factors** | **Failures** | **Event rate per 1000 patient-years**  **(95% Cl)** | **Unadjusted** | | **Model 1** | | **Model 2** | | **Model 3** | |
| --- | --- | --- | --- | --- | --- | --- | --- | --- | --- | --- |
|  |  |  | **HR (95% Cl)** | **P value** | **HR (95% Cl)** | **P value** | **HR (95% Cl)** | **P value** | **HR (95% Cl)** | **P value** |
| All-cause graft failure* | | | | | | | | | | |
| Donor AKI stage |  |  |  | 0.05 |  | 0.03 |  | 0.04 |  | 0.09 |
| No AKI (n=8919) | 2857 | 49.7 (47.9- 51.5) | Ref |  | Ref |  | Ref |  | Ref |  |
| Stage 1 (n=693) | 271 | 60.2 (53.4- 67.8) | 1.21 (1.06- 1.39) | <0.01 | 1.22 (1.06- 1.40) | <0.01 | 1.20 (1.05-1.39) | <0.01 | 1.18 (1.03-1.37) | 0.02 |
| Stage 2 (n=264) | 79 | 48.0 (38.5- 59.8) | 0.96 (0.75- 1.23) | 0.75 | 0.96 (0.74- 1.23) | 0.73 | 0.94 (0.74- 1.22) | 0.69 | 0.92 (0.71- 1.18) | 0.49 |
| Stage 3 (n=225) | 52 | 52.8 (40.2- 69.3) | 1.09 (0.82- 1.43) | 0.56 | 1.18 (0.88-1.58) | 0.27 | 1.17 (0.88-1.58) | 0.28 | 1.13 (0.83- 1.54) | 0.43 |
| Death-censored graft failure* | | | | | | | | | | |
| Donor AKI stage |  |  |  | 0.58 |  | 0.32 |  | 0.37 |  | 0.38 |
| No AKI (n=8919) | 1540 | 26.8 (25.5- 28.1) | Ref |  | Ref |  | Ref |  | Ref |  |
| Stage 1 (n=693) | 136 | 30.2 (25.5- 35.7) | 1.13 (0.94- 1.37) | 0.20 | 1.15 (0.95- 1.38) | 0.15 | 1.13 (0.94- 1.37) | 0.20 | 1.11 (0.92- 1.35) | 0.27 |
| Stage 2 (n=264) | 44 | 26.7 (19.9- 35.9) | 0.99 (0.71- 1.38) | 0.96 | 0.96 (0.67-1.36) | 0.82 | 0.95 (0.67- 1.35) | 0.78 | 0.92 (0.63- 1.33) | 0.65 |
| Stage 3 (n=225) | 30 | 30.5 (21.3- 43.6) | 1.11 (0.78- 1.59) | 0.55 | 1.27 (0.87- 1.85) | 0.22 | 1.26 (0.86- 1.88) | 0.23 | 1.31 (0.87- 1.97) | 0.19 |
| All-cause mortality* | | | | | | | | | | |
| Donor AKI stage |  |  |  | 0.08 |  | 0.09 |  | 0.11 |  | 0.15 |
| No AKI (n=8919) | 1405 | 24.4 (23.2- 25.7) | Ref |  | Ref |  | Ref |  | Ref |  |
| Stage 1 (n=693) | 140 | 31.1 (26.4- 36.7) | 1.27 (1.06- 1.53) | 0.01 | 1.27 (1.06- 1.53) | 0.01 | 1.30 (1.05- 1.52) | 0.02 | 1.25 (1.03-1.51) | 0.02 |
| Stage 2 (n=264) | 39 | 23.7 (17.3- 32.4) | 0.97 (0.69- 1.36) | 0.84 | 1.00 (0.71- 1.41) | 1.00 | 0.99 (0.71-1.40) | 0.98 | 0.98 (0.70-1.36) | 0.89 |
| Stage 3 (n=225) | 24 | 24.4 (16.3- 39.7) | 1.06 (0.70-1.60) | 0.78 | 1.12 (0.71- 1.74) | 0.63 | 1.11 (0.71- 1.73) | 0.64 | 0.96 (0.60- 1.53) | 0.85 |
|  |  |  | sHR (95% CI) |  | sHR (95% CI) |  | sHR (95% CI) |  | sHR (95% CI) |  |
| Graft failure with death as a competing event^#^ | | |  |  |  |  |  |  |  |  |
| Donor AKI stage |  |  |  | 0.81 |  | 0.52 |  | 0.59 |  | 0.54 |
| No AKI (n=8919) | 1540 | 26.8 (25.5- 28.1) | Ref |  | Ref |  | Ref |  | Ref |  |
| Stage 1 (n=693) | 136 | 30.2 (25.5- 35.7) | 1.09 (0.91-1.32) | 0.99 | 1.11 (0.93- 1.32) | 0.26 | 1.10 (0.91-1.31) | 0.34 | 1.08 (0.90- 1.31) | 0.41 |
| Stage 2 (n=264) | 44 | 26.7 (19.9- 35.9) | 1.00 (0.72-1.40) | 0.71 | 0.97 (0.71- 1.34) | 0.86 | 0.97 (0.70- 1.33) | 0.85 | 0.93 (0.64- 1.35) | 0.71 |
| Stage 3 (n=225) | 30 | 30.5 (21.3-43.6) | 1.07 (0.75-1.52) | 0.78 | 1.22 (0.83- 1.78) | 0.31 | 1.21 (0.83- 1.77) | 0.31 | 1.28 (0.84- 1.94) | 0.24 |

Data presented as HR with 95% confidence interval (95%Cl) from Cox regression models (^*^ for all cause graft failure, death-censored graft failure and all-cause mortality) or as sub-distribution (sHR) with 95%CI from competing risk model (^#^ for graft failure with death as competing event).

Model 1:  AKI (KDIGO definition) + KDRI components except for terminal SCr (already considered for the AKI covariate), Model 2:  Model 1 + other donor factors such as sex, number of individual kidneys transplanted, Model 3:  Model 2 + recipient and transplant factors, which included age (years), gender, ethnicity, BMI, Previous transplant, Pre-emptive transplant, Cause of ESKD, Dialysis vintage, Anti-rejection immunosuppression, HLA-mismatches number, Peak panel reactive antibody (%), total ischemia time (hours) and era. Model 1: AKI (KDIGO definition) + KDRI components except for terminal SCr (already considered for the AKI covariate), Model 2: Model 1 + other donor factors such as sex, number of kidneys eventually donated, Model 3: Model 2 + recipient and transplant factors, which included age, sex, ethnicity, body mass index [BMI], previous transplant, pre-emptive transplant, cause of ESKD, dialysis vintage, induction immunosuppression, number of human leukocyte antigen-mismatches [HLA-A, HLA-B, HLA-DR], peak panel reactive antibody, total ischemia time, era [1997-2003, 2004-2010, 2011-2017]

Abbreviations: AKI, acute kidney injury; HR, hazard ratio; Cl, confidence interval; sHR, sub-distribution hazard ratio.
